# A central role for canonical PRC1 in shaping the 3D nuclear landscape

**DOI:** 10.1101/gad.336487.120

**Published:** 2020-07-01

**Authors:** Shelagh Boyle, Ilya M. Flyamer, Iain Williamson, Dipta Sengupta, Wendy A. Bickmore, Robert S. Illingworth

**Affiliations:** MRC Human Genetics Unit, Institute of Genetics and Molecular Medicine, University of Edinburgh, Edinburgh EH4 2XU, United Kingdom

**Keywords:** polycomb, topologically associating domains (TADs), gene repression, nuclear organization, embryonic stem cells, gene regulation, epigenetics, histone modifications

## Abstract

Polycomb group (PcG) proteins silence gene expression by chemically and physically modifying chromatin. A subset of PcG target loci are compacted and cluster in the nucleus; a conformation that is thought to contribute to gene silencing. However, how these interactions influence gross nuclear organization and their relationship with transcription remains poorly understood. Here we examine the role of Polycomb-repressive complex 1 (PRC1) in shaping 3D genome organization in mouse embryonic stem cells (mESCs). Using a combination of imaging and Hi-C analyses, we show that PRC1-mediated long-range interactions are independent of CTCF and can bridge sites at a megabase scale. Impairment of PRC1 enzymatic activity does not directly disrupt these interactions. We demonstrate that PcG targets coalesce in vivo, and that developmentally induced expression of one of the target loci disrupts this spatial arrangement. Finally, we show that transcriptional activation and the loss of PRC1-mediated interactions are separable events. These findings provide important insights into the function of PRC1, while highlighting the complexity of this regulatory system.

## Abstract (precis)

In this study from Boyle et al., the authors investigated the role of Polycomb-repressive complex 1 (PRC1) in shaping 3D genome organization in mouse embryonic stem cells. Using a combination of imaging and Hi-C analyses they show that PRC1-mediated long-range interactions are independent of CTCF and can bridge sites at a megabase scale, thus providing novel insights into the function of PRC1.

The spatial and temporal fidelity of development is controlled by transcription factors that act in concert with the epigenome to regulate gene expression programs (Simon and Kingston 2013; Atlasi and Stunnenberg 2017). Polycomb repressive complexes (PRCs), a family of essential epigenetic regulators, modify chromatin to propagate a repressed but transcriptionally poised state (Brookes and Pombo 2009; Simon and Kingston 2013; Voigt et al. 2013; Blackledge et al. 2015; Schuettengruber et al. 2017). PRC1 and PRC2, the two principal members of this family, prevent unscheduled differentiation by targeting and restricting the expression of genes encoding key developmental regulators. The deposition of H2AK119ub1 and H3K27me1/2/3 by RING1A/B (PRC1) and EZH1/2 (PRC2), respectively, is required for the efficient placement of PRCs at target loci (Poux et al. 2001; Cao et al. 2002; Czermin et al. 2002; Kuzmichev et al. 2002; Müller et al. 2002; de Napoles et al. 2004; Wang et al. 2004a,b; Margueron et al. 2009; Blackledge et al. 2014; Cooper et al. 2014; Kalb et al. 2014).

The core of PRC1 comprises a heterodimer of RING1A/B and one of six PCGF RING finger proteins. Deposition of H2AK119Ub is driven primarily by variant PRC1s (vPRC1–RING1A/B complexed with either PCGF1, PCGF3, PCGF5, or PCGF6), which have enhanced E3 ligase activity due to an association with either RYBP or YAF2 (Rose et al. 2016; Fursova et al. 2019). Combinatorial deletion of PCGF1, PCGF3, PCGF5, and PCGF6 in mouse embryonic stem cells (mESCs) leads to substantial gene misregulation and highlights the importance of vPRC1s in transcriptional control (Fursova et al. 2019). In contrast, canonical PRC1 (cPRC1; core heterodimer of RING1A or RING1B with PCGF2 or PCGF4) has lower catalytic activity and is instead associated with subunits that alter chromatin structure and topology (Francis et al. 2004; Grau et al. 2011; Isono et al. 2013; Blackledge et al. 2014; Taherbhoy et al. 2015; Wani et al. 2016; Kundu et al. 2017; Lau et al. 2017; Plys et al. 2019; Tatavosian et al. 2019). In line with this function, a subset of PRC1 targets fold into short discrete self-interacting domains (20–140 kb), exemplified by the conformation of the transcriptionally silent *Hox* gene clusters in mESCs (Eskeland et al. 2010; Williamson et al. 2014; Vieux-Rochas et al. 2015; Kundu et al. 2017). However, unlike topologically associated domains (TADs), which are somewhat structurally invariant across different cells types, PRC1-mediated domains are developmentally dynamic and are eroded upon gene activation and the loss of PRC1 association (Lieberman-Aiden et al. 2009; Eskeland et al. 2010; Dixon et al. 2012; Nora et al. 2012; Williamson et al. 2012; Rao et al. 2014; Bonev et al. 2017; Kundu et al. 2017). In addition to local chromatin folding, PRC1 coordinates interactions between distally located target sites (Isono et al. 2013; Schoenfelder et al. 2015; Kundu et al. 2017). Consequently, genomic loci that are separated by large distances in the linear genome can be brought into close spatial proximity. In *Drosophila*, this juxtaposition has been suggested to enhance transcriptional repression, but direct evidence for this in mammalian cells is lacking (Bantignies et al. 2003, 2011; Eagen et al. 2017; Ogiyama et al. 2018).

CBX and PHC subunits are thought to be the components of PRC1 that are primarily responsible for mediating these chromatin structures. CBX2, a mammalian homolog of *Drosophila* Polycomb, contains a positively charged intrinsically disordered region (IDR) that can compact nucleosomal arrays in vitro (Grau et al. 2011). Neutralizing amino acid substitutions in the IDR of CBX2 leads to some loss of PRC1-mediated gene repression and axial patterning defects in mice (Lau et al. 2017). Not all CBX subunits possess this function (including CBX4 and CBX7); however, those with the capacity to alter chromatin structure (CBX2, CBX6, and CBX8) account for approximately half of all PRC1 in mESCs (Grau et al. 2011; Kloet et al. 2016). Polyhomoeotic (PHC) proteins can make both homomeric and heteromeric head-to-tail interactions via their sterile α motif (SAM) domain (Isono et al. 2013), allowing multiple cPRC1s to oligomerize and thus to physically connect regions of the genome. Disruption of the SAM domain ablates these interactions, leading to the loss of both local interaction domains and PRC1 mediated looping (Kundu et al. 2017) and resulting in gene derepression and skeletal abnormalities in mice (Isono et al. 2013). Furthermore, loss of these architectural PRC1 subunits leads to the dissolution of nanometre scale “polycomb bodies” containing high local concentrations of polycomb proteins (Isono et al. 2013; Wani et al. 2016; Plys et al. 2019; Tatavosian et al. 2019). These data support the idea that CBX and PHC proteins bestow cPRC1 with the capacity to fold chromatin into discrete nuclear domains and suggest a mechanistic role for chromatin interactions and nuclear clustering in PRC1-mediated transcriptional repression.

However, this emerging view raises some important questions. What factors determine which distal PRC1 targets will physically interact? Does PRC1 create a topology that anchors multiple loci simultaneously in a single cell and, if so, do such structures occur in vivo? What is the cause/consequence relationship between chromatin structure and gene derepression in cells lacking RING1B? In this study, we used both Hi-C and DNA Fluorescence in Situ Hybridization (FISH) in mESCs and embryonic mouse tissue to investigate how PRC1 contributes to nuclear organization. We find that PRC1 has a substantial effect on chromosomal architecture that is disproportionate to the fraction of the genome it occupies. These structures rely on canonical PRC1, are independent of CTCF, and persist even when RING1B catalytic activity is substantially impaired. Our findings provide key insights into the manner in which PRC1 directs the 3D topology of the mammalian genome.

## Results

### Loss of RING1B disrupts nuclear clustering of Polycomb targets

**Figure 1.**
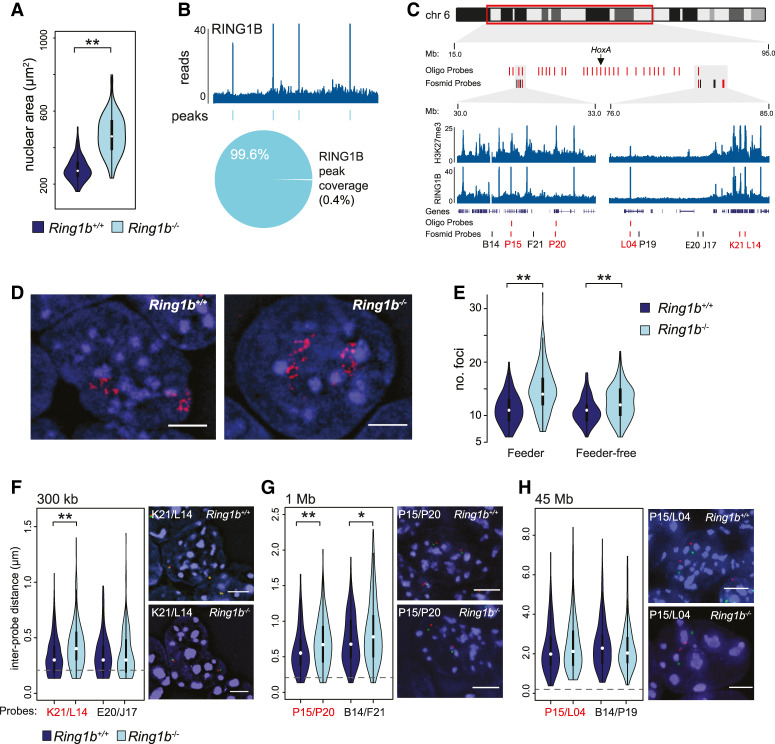
PRC1 dependent nuclear clustering of polycomb targets. (*A*) Violin plot depicting the nuclear area (μm^2^) of *Ring1b*^+/+^ and *Ring1b*^−/−^ mESCs determined by DAPI staining of 2D preparations. (**) *P* = 9.25 × 10^−23^; Mann Whitney *U*-test. (*B*) Example ChIP-seq profile and called peaks for RING1B in wild-type mESCs (Chr 6: 62.5- to 68.5-Mb mm9 genome assembly) (Illingworth et al. 2015). The pie chart *below* shows the sum coverage of all RING1B peaks as a fraction of the uniquely mappable portion of the mouse genome. (*C*) Ideogram of chromosome 6 showing the location of oligonucleotide and fosmid probes used in *D*–*H* and zoomed browser tracks of RING1B and H3K27me3 ChIP-seq from wild-type mESCs (Illingworth et al. 2015). Genome coordinates from the mm9 genome assembly. (*D*) Representative 3D FISH image of the chromosome 6 polycomb positive oligonucleotide probe signal in the nuclei of *Ring1b*^+/+^ and *Ring1b*^−/−^ mESCs. Scale bar, 5 μm. (*E*) Violin plot depicting the number of discrete foci in *Ring1b*^+/+^ and *Ring1b*^−/−^ mESCs detected by FISH with the chromosome 6 polycomb-positive oligonucleotide probe. Two independent *Ring1b*^−/−^ clones and their associated wild-type parental mESCs are indicated (“feeder” and “feeder-free”) (Leeb and Wutz 2007; Illingworth et al. 2015). (**) *P* = 9.07 × 10^−17^ for feeder; (**) *P* = 5.33 × 10^−6^ for feeder-free; Mann Whitney test. (*F*–*H*) Violin plots of interprobe distances (μm) for the indicated fosmids (locations shown in *C* with representative images for both *Ring1b*^+/+^ and *Ring1b*^−/−^ mESCs). Scale bar, 5 μm. Probes separated by <0.2 μm (dashed gray line) are considered to be colocalized. (**) *P* = 4.23 × 10^−07^ in *F*; (**) *P* = 9.47 × 10^−04^; (*) *P* = 3.47 × 10^−02^ in *G*; Mann Whitney test.

RING1B is the primary RING1 homolog expressed in mESCs, and in its absence levels of the PRC1 complex are substantially reduced (Leeb and Wutz 2007; Endoh et al. 2008; Eskeland et al. 2010). DAPI staining of 2D nuclear preparations revealed a significant increase in nuclear area in the absence of PRC1 (*Ring1b*^−/−^) when compared with parental *Ring1b*^+/+^ mESCs (Fig. 1A; Supplemental Fig. S1A). The polycomb system promotes cell proliferation, in part, by negatively regulating inhibitors of the cell cycle (Jacobs et al. 1999; Gil et al. 2004; Bracken et al. 2007; Chen et al. 2009). However, fluorescence-activated cell sorting (FACS) did not identify an altered cell cycle profile between *Ring1b*^+/+^ and *Ring1b*^−/−^ cells (Supplemental Fig. S1B). This suggested that the increase in nuclear size in *Ring1b*^−/−^ cells is a direct consequence of PRC1 depletion on nuclear structure, rather than an accumulation of cells in G2.

Canonical PRC1 can directly alter local chromatin structure, and chromatin conformation capture assays (e.g., Hi-C) have demonstrated that polycomb target sites can physically interact (Eskeland et al. 2010; Williamson et al. 2014; Joshi et al. 2015; Schoenfelder et al. 2015; Vieux-Rochas et al. 2015; Bonev et al. 2017; Kundu et al. 2017). However, analysis of ChIP-seq data indicates that only a very small fraction (0.4%) of the mESC genome has pronounced RING1B occupancy (Fig. 1B; Illingworth et al. 2015). How then could the loss of RING1B/PRC1 at discrete sites lead to such a profound impact on nuclear size? To address this question, we determined the spatial arrangement of polycomb (PcG) target loci within individual nuclei and how this was disrupted in cells lacking RING1B. For this we needed a probe for 3D-FISH that would simultaneously detect multiple PcG target loci. We therefore designed a custom fluorescently labeled oligonucleotide pool covering 30 noncontiguous 20-kb windows along chromosome 6, each of which was centered on an individual PcG target locus (H3K27me3 positive peaks from ChIP-seq data; (Fig. 1C; Supplemental Table S1; Illingworth et al. 2015). Hybridization to metaphase spreads confirmed the specificity and efficiency of the oligonucleotide probe (Supplemental Fig. S1C). In interphase, quantitation of foci by blind scoring demonstrated a marked colocalization of polycomb targets. A median score of six foci per chromosome suggested that at least five or more PcG target sites might simultaneously localize to a single focus (Fig. 1D,E). The mean number of foci detected was significantly increased in each of two independent *Ring1b*^−/−^ mESC lines (*P* = 9.1 × 10^−17^ for feeder-dependent and *P* = 5.3 × 10^−6^ for feeder-independent ESC lines, respectively) (Fig. 1E; Leeb and Wutz 2007; Illingworth et al. 2015), suggesting that polycomb targets cluster together in a PRC1-dependent manner in mESCs.

It has been shown that polycomb-mediated interactions can occur over large genomic distances (>10 Mb) (Joshi et al. 2015; Schoenfelder et al. 2015; Vieux-Rochas et al. 2015; Bonev et al. 2017; Kundu et al. 2017). To determine whether polycomb site clustering was restricted by the linear proximity of target sites along the genome we performed 3D-FISH using fluorescently labeled fosmid probes targeting polycomb-positive (PcG^+^) and polycomb-negative (PcG^−^) loci (presence or absence of H3K27me3, respectively) located along the same region of chromosome 6 (Fig. 1C). PcG^+^ sites relatively close to each other in the linear genome (300 kb and 1 Mb) showed reduced colocalization, and a significant increase in interprobe distances in cells lacking RING1B (Fig. 1F,G; Supplemental Fig. S1D,E; FISH signals separated by <0.2 μm (Fig. 1F,G, dashed gray line) are considered to be colocalized). In contrast, loss of RING1B did not significantly impact on the interprobe distances when the probes were separated by 45 Mb (Fig. 1H; Supplemental Fig. S1F). These findings suggest that although long-range sites can be detected in close proximity within a population of cells, PRC1-mediated associations are generally constrained, either by linear separation along the DNA fibre or by local chromosomal topology. Interestingly, PcG^−^ “control” probes separated by 1 Mb also showed a significant increase in interprobe distance in *Ring1b*^−/−^ mESCs despite lacking detectible H3K27me3 or RING1B (Fig. 1C,H; Supplemental Fig. S1E). This suggests that the influence of PRC1 on chromosomal topology extends beyond just those sites immediately bound by polycomb proteins and could explain the increase in nuclear size in the absence of RING1B (Fig. 1A).

### PRC1-mediated interactions have a profound influence on gross nuclear organization

To investigate the possibility that PRC1-mediated interactions influence the topology of intervening chromatin, we interrogated the spatial proximity of additional genomic sites that lacked detectible H3K27me3 and RING1B signal. We performed 3D-FISH using pairs of fosmid probes spaced 300-kb apart and located ∼0.5 Mb from the nearest RING1B peak (Supplemental Fig. S2A). In line with our previous observation, two independent loci showed a significant increase in interprobe distances in *Ring1b*^−/−^ mESCs compared with *Ring1b*^+/+^ controls (Fig. 1G; Supplemental Fig. S1E); Supplemental Fig. S2A). However, we noted no such effect for a negative probe pair on chromosome 6 (Fig. 1F; Supplemental Fig. S1D). Consequently, analysis of single sites in this manner provided only limited insight into the impact of RING1B loss on PRC1 bound and nonbound chromatin.

**Figure 2.**
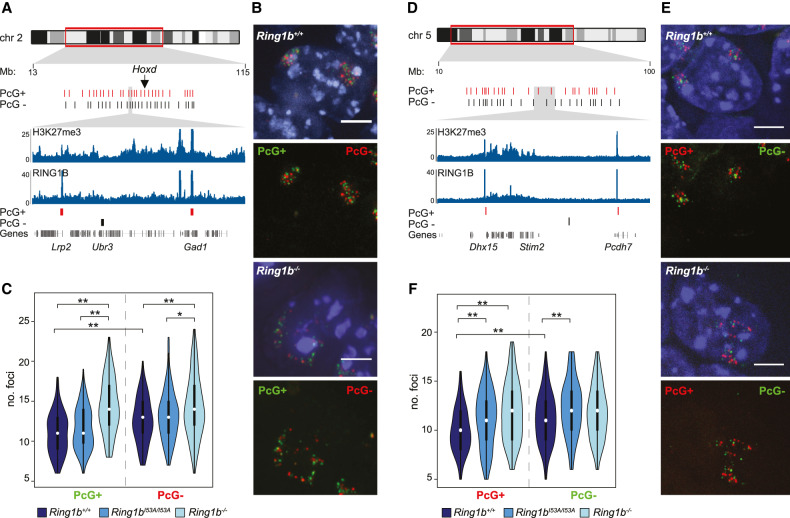
Loss of PRC1 reduces nuclear clustering at sites with and without RING1B. (*A*) Ideogram of chromosome 2 indicating the location of the oligonucleotide probes used in *B* and *C* and zoomed in browser tracks of RING1B and H3K27me3 ChIP-seq from wild-type mESCs (Illingworth et al. 2015). Polycomb-positive (PcG^+^) and polycomb-negative (PcG^−^) are represented as red and black bars, respectively. Genomic locations are for the mm9 genome assembly. (*B*) Representative 3D FISH images of *Ring1b*^+/+^ and *Ring1b*^−/−^ mESCs hybridized with the chromosome 2 oligonucleotide PcG^+^ (green; 6FAM) and PcG^−^ (red; ATT0594) probes. Scale bar = 5 μm. (*C*) Violin plots depicting the number of discrete foci in *Ring1b*^+/+^, *Ring1b^I53A/I53A^*, and *Ring1b*^−/−^ mESCs detected by the PcG^+^ and PcG^−^ FISH probe pools. (*) *P* ≤ 0.05; (**) *P* ≤ 0.01; Mann Whitney test. (*D*–*F*) As for *A–C*, but for a second set of oligonucleotide probes targeted to chromosome 5. (*E*) PcG^+^ (red; ATT0594) and PcG^−^ (green; 6FAM).

To address this, we used oligonucleotide probe pools to investigate more extensively the impact of RING1B loss on the 3D arrangement of loci with and without PRC1 occupancy. We designed two sets of probe pools covering 28 discrete loci along a 51-Mb portion of chromosome 2. The polycomb-positive probe (PcG^+^) was targeted to H3K27me3 “peaks” enriched for RING1B occupancy (Fig. 2A - red bars; Supplemental Table 1). The nonpolycomb probe (PcG^−^) was designed against nonrepetitive sites lacking detectible H3K27me3 or RING1B ChIP-seq signal and was offset in the linear genome relative to the regions covered by the PcG^+^ probe (Fig. 2A, black bars; Supplemental Table S1). 3D-FISH of *Ring1b*^+/+^ mESCs cohybridized with these two probe sets showed that the PcG^−^ sites were significantly less clustered than those marked by the PcG^+^ probe (Fig. 2B,C; Supplemental Fig. S2B). As expected, loss of RING1B led to a significant loss of clustering between PcG^+^ loci but also a reduction, albeit more subtle, in the clustering of the intervening non-polycomb sites (Fig. 2B,C; Supplemental Fig. S2B). Compatible with our earlier observations, this suggested that reduced PRC1 levels in *Ring1b*^−/−^ mESCs impacts the conformation, not only of those sites directly associated with polycomb, but of the intervening chromatin also.

Strikingly, mESCs bearing a homozygous mutation encoding an isoleucine to alanine substitution at amino acid 53 of RING1B (*Ring1b^I53A/I53A^*) that profoundly impairs its E3 ubiquitin ligase activity but preserves the integrity of the PRC1 complex, (Buchwald et al. 2006; Eskeland et al. 2010; Illingworth et al. 2010, 2015; Endoh et al. 2012; Pengelly et al. 2015) did not disrupt clustering between either of the sets of loci being interrogated (Fig. 2C). This suggests that, as for local chromatin compaction (Eskeland et al. 2010), the catalytic activity of RING1B does not directly contribute to its ability to alter chromatin architecture and supports the notion that chromatin architecture and histone ubiquitination are functionally separable (Isono et al. 2013; Taherbhoy et al. 2015; Rose et al. 2016; Kundu et al. 2017; Fursova et al. 2019). However, recent insights have highlighted the importance of ncPRC1-mediated H2AK119Ub in the targeting of cPRC1 (Blackledge et al. 2014; Cooper et al. 2014; Rose et al. 2016; Fursova et al. 2019). Therefore, we reasoned that some genomic regions might be more sensitive to impaired RING1B catalytic activity and this might have a secondary influence on chromatin architecture and nuclear organization. To investigate this we used an oligonucleotide probe set to target a region that displayed a more pronounced loss of RING1B binding in *Ring1b^I53A/I53A^* mESCs than that observed for the chromosome 2 region discussed above (Fig. 2A–C; Supplemental Fig. S2C; Illingworth et al. 2015). These probe-sets spanned ∼60 Mb of chromosome 5 and covered 25 discrete loci (Fig. 2D; Supplemental Table S1). As for chromosome 2, the PcG^+^ sites were significantly more clustered than a set of intervening sequences that lacked discernible polycomb signal (H3K27me3 and RING1B) (Fig. 2E,F; Supplemental Fig. S2D). Polycomb-positive and polycomb-negative sites also showed reduced clustering in cells lacking RING1B but, as for chromosome 2, the effect on the PcG^−^ sites was more subtle with only one of the two experiments yielding a significant reduction in clustering (*P* = 0.09 and *P* = 0.04, respectively) (Fig. 2F; Supplemental Fig. S2D). Interestingly, unlike chromosome 2, clustering of both PcG^+^ and PcG^−^ sites within this region of chromosome 5 was substantially impaired in the catalytically deficient RING1B mutant (*Ring1b^I53A/I53A^*) mESCs in line with a more pronounced reduction in RING1B occupancy in this region in these cells (Fig. 2F; Supplemental Fig. S2C).

Taken together, these findings suggest that PRC1 influences gross nuclear organization by altering not only the polycomb-bound portion of the genome but also by affecting the conformation of intervening chromatin. Furthermore, RING1B-mediated ubiquitination does not directly contribute to polycomb-dependent nuclear clustering, yet its loss indirectly disrupts the association of sites where RING1B binding is substantially reduced.

### Genome-wide compaction of polycomb targets

To investigate what influence PRC1 binding has on 3D organization genome-wide, we performed in situ Hi-C on *Ring1b*^+/+^, *Ring1b^I53A/I53A^*, and *Ring1b*^−/−^ mESCs to obtain a total of ∼300 million contacts >1 kb. Given the dramatic changes in nuclear size, and chromatin organization observed by FISH, we were surprised to find that the dependency of contact probability on genomic distance was largely unaffected by *Ring1b* mutations (Supplemental Fig. S3A). Next we analyzed the impact of *Ring1b* mutations on A/B compartmentalization using eigenvector decomposition. At this scale (200 kb), *Ring1b*^+/+^ and *Ring1b^I53A/I53A^* were highly similar, while *Ring1b*^−/−^ mESCs showed a more distinct 3D genome organization, both by clustering and principal component analysis (PCA; Supplemental Fig. S3B,C). A and B compartmentalization reflects the spatial segregation of active and inactive chromatin in the nucleus; therefore, this result likely reflects the more pronounced transcriptional changes in *Ring1b*^−/−^ compared with *Ring1b^I53A/I53A^* mESCs (Illingworth et al. 2015). To directly test this possibility, we compared gene expression levels with regional compartment scores (50-kb resolution) in wild-type versus mutant (*Ring1b*^−/−^ or *Ring1b^I53A/I53A^*) mESCs. As expected, alterations in compartment score associated with, and was proportionate to, changes in gene expression between *Ring1b*^+/+^, *Ring1b^I53A/I53A^*, and *Ring1b*^−/−^ mESC lines (Supplemental Fig. S3D).

**Figure 3.**
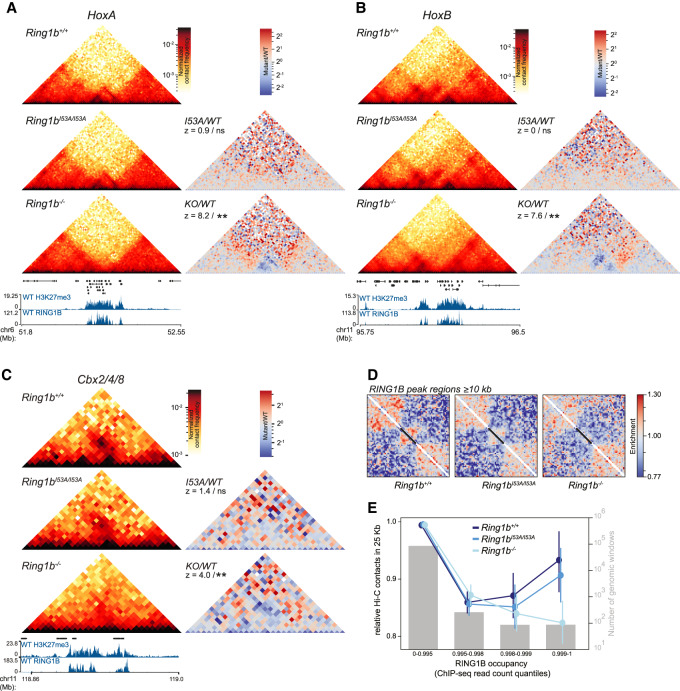
Local compaction of PRC1 targets. (*A*) Hi-C maps of the *HoxA* cluster from *Ring1b^+/+^, Ring1b^I53A/I53A^*, and *Ring1b*^−/−^ mESCs at 10-kb resolution. Ratios of maps from mutant over wild-type cells also shown at the *right* together with statistical estimation (*z*-score and significance level; [ns] *P* > 0.05; [**] *P* ≤ 0.01) of the difference in contact frequency within the *HoxA* cluster (statistical estimation performed on chr6 52.1- to 52.22-Mb mm9 genome build) between the cell lines. Genes, H3K27me3 and RING1B ChIP-seq profiles are shown below. (*B*) Same as *A*, but for *HoxB* (statistical estimation performed on chr11 96.11–96.22 Mb). (*C*) As in *A*, but for *Cbx2/4/8* (statistical estimation performed on chr11 118.88–118.96 Mb; mm9 genome build). (*D*) Rescaled observed/expected pileups of all RING1B peak regions ≥10 kb in length (*n* = 181) in *Ring1b*^+/+^, *Ring1b^I53A/I53A^*, and *Ring1b*^−/−^ cells. Black bars represent the location of the RING1B peak regions in the averaged map. (*E*) Average (±95% confidence interval) level of observed/expected contacts within 25-kb genomic windows split by percentiles of wild-type RING1B ChIP-seq signal shown for each of *Ring1b^+/+^, Ring1b^I53A/I53A^*, and *Ring1b*^−/−^ mESCs. Gray bars show number of windows in each category (*right Y*-axis).

5C analysis of candidate loci has demonstrated that chromosomal regions with high local PRC1 occupancy are folded into a discrete self-interacting configuration (Noordermeer et al. 2011; Williamson et al. 2014; Kundu et al. 2017). To investigate local compaction in our Hi-C data, we first focused on the *Hox* loci; the most extended polycomb-associated loci in the mouse genome. These regions have previously been shown to be highly compacted by polycomb complexes, and to lose this compaction upon loss of PRC1 (Eskeland et al. 2010; Williamson et al. 2014; Kundu et al. 2017). Consistent with those observations, we detect regions of high interaction frequency inside the *Hox* clusters, that are completely lost in *Ring1b*^−/−^ mESCs (Fig. 3A,B; Supplemental Fig. S3E). *Ring1b^I53A/I53A^* mESCs did not display a significant loss of interactions relative to *Ring1b*^+/+^ mESCs (Fig. 3A,B; Supplemental Fig. S3E) suggesting only a modest disruption of PRC1-dependent 3D organization in cells bearing this mutation, consistent with our FISH data.

To investigate whether this was restricted to *Hox* clusters or was a more general property of extended regions of polycomb binding, we inspected other regions of pronounced polycomb association, including the *Cbx2(4/8)* and *Nr2f2* loci (Fig. 3C; Supplemental Fig. S3F). While these regions are substantially shorter than the *Hox* clusters, we observed a similar high local contact frequency in both *Ring1b*^+/+^ and *Ring1b^I53A/I53A^* mESCs that was lost in *Ring1b*^−/−^ cells (Fig. 3C; Supplemental Fig. S3F). To investigate PRC1-mediated compaction genome-wide, we performed local pile-up analysis of all 181 extended RING1B ChIP-seq peak regions (≥10 kb) using 1-kb resolution Hi-C data. We rescaled each region of the Hi-C map to the same length and compared the resulting pileups between each of the three mESC cell lines. Consistent with our candidate analysis, *Ring1b*^+/+^ cells demonstrated a clear enrichment of local interactions corresponding to the extent of RING1B occupancy (Fig. 3D). This enrichment was subtly lower in *Ring1B^I53A/I53A^* cells, and completely absent in the Ring1B^−/−^ cells (Fig. 3D). As an alternative validation of this result, we grouped all 25-kb genomic windows into quantiles of RING1B occupancy (ChIP-seq) and compared the mean observed/expected Hi-C contacts for each of these groups in each of the three mESC lines. Genomic windows bearing the highest RING1B occupancy had a local contact frequency that was substantially higher in *Ring1b*^+/+^ cells than in *Ring1b*^−/−^, but *Ring1b^I53A/I53A^* were only mildly affected in line with our own and published observation (Fig. 3E; Supplemental Fig. S3G; Kundu et al. 2017). These findings suggest that local interaction domains are a characteristic property of extended chromosomal regions bearing high levels of PRC1 binding and that this is largely independent of the catalytic activity of PRC1.

### High levels of canonical PRC1 drive distal interactions independently of CTCF

**Figure 4.**
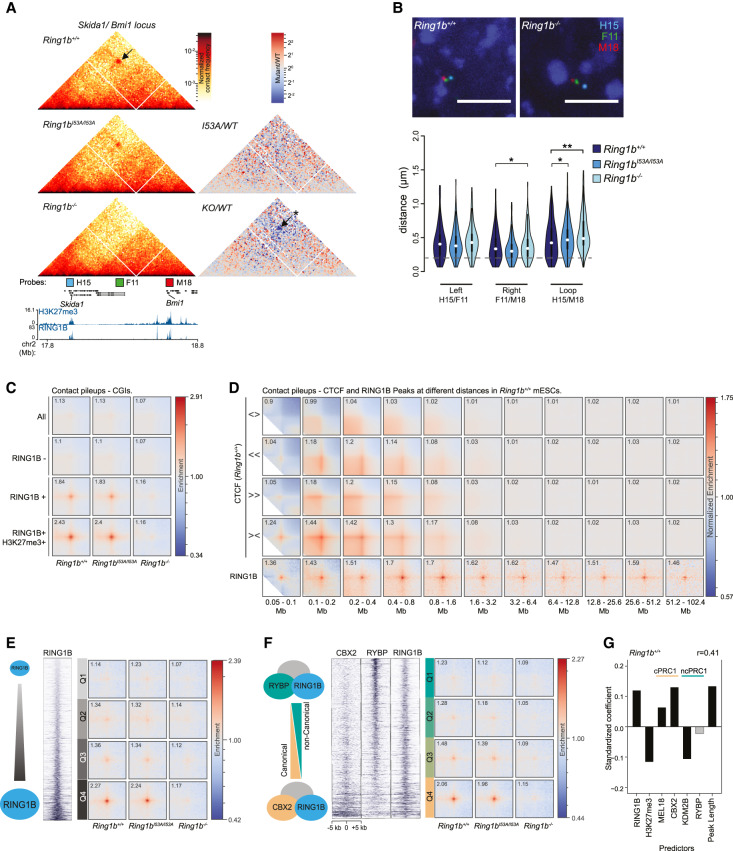
Characterization of distal interactions between PRC1 targets. (*A*) Hi-C data for the region of chromosome 2 harboring the *Skida1* and *Bmi1* polycomb targets. Data presented as for Figure 3A. Distal interactions are highlighted with arrows and the significance of differntial signal between *Ring1b*^+/+^ and *Ring1b*^−/−^ Hi-C data is indicated. (*) *P* ≤ 0.05 and *P* > 0.01. Also shown are the locations of FISH probes for the PcG^+^ *Skida1* (H15) and *Bmi1* (M18) loci and an intervening PcG^−^ site (F11). (*B*) Representative images of 3D FISH from *Ring1b*^+/+^ and *Ring1b*^−/−^ cells with probes shown in *A*. (*Below*) Violin plots show the interprobe distances (µm) for probe pairs shown in *A* in *Ring1b^+/+^, Ring1b^I53A/I53A^*, and *Ring1b*^−/−^ cells. (*) *P* ≤ 0.05 and *P* > 0.01; (**) *P* ≤ 0.01; Mann Whitney test. Probes separated by <0.2 μm (dashed gray line) are considered to be colocalized. (*C*) Pileups of interactions between CGIs. In rows: all CGIs, RING1B-negative CGIs, RING1B-positive CGIs, and RING1B/H3K27me3-double-positive CGIs. In columns: *Ring1b^+/+^, Ring1b^I53A/^*^I53A^*,* and *Ring1b*^−/−^ cells. (*D*) Pileups of interactions between CTCF sites and RING1B peaks at different distance separations for *Ring1b*^+/+^ cells. In rows: divergent CTCF sites, left-facing CTCF sites, right-facing CTCF sites, convergent CTCF sites, and RING1B peaks. In columns: twofold increasing distance separation ranges from 0.05–0.1 Mb to 51.2–102.4 Mb. (*E*) Pileups for RING1B peaks with different level of RING1B binding by ChIP-seq. In rows: four quartiles of RING1B occupancy. In columns: *Ring1b^+/+^, Ring1b^I53A/I53A^*, and *Ring1b*^−/−^ cells. (*F*) Same as *E*, but for quartiles of CBX2/RYBP ratio instead of RING1B occupancy. (*G*) Linear model coefficients for prediction of loop ability of RING1B peaks for *Ring1b*^+/+^ cells based on properties of RING1B peak regions (*X*-axis). Positive values indicate positive impact on loopability. Light gray bars are not significant. *P*-value > 0.05. Pearson's correlation coefficient of predicted versus observed values is shown at the *top right*. Predictors associated with canonical and noncanonical PRC1 are denoted by “c” and “nc,” respectively.

Beyond the scale of local interaction domains, chromatin conformation capture assays have demonstrated that distal polycomb target sites can interact and loop together into close spatial proximity (Joshi et al. 2015; Schoenfelder et al. 2015; Vieux-Rochas et al. 2015; Bonev et al. 2017; Kundu et al. 2017; McLaughlin et al. 2019). Hi-C interactions between distal PRC1-binding sites were evident in our data and, as for local compaction, were largely preserved in *Ring1b^I53A/I53A^* mESCs but completely lost in cells lacking RING1B (Fig. 4A; Supplemental Fig. S4A,B). To validate the interactions observed between *Bmi1* and *Skida1* (∼600 kb) we performed four-color FISH with three fosmid probes targeting both of these PcG^+^ gene loci and the intervening PcG^−^ midpoint (Fig. 4A,B; Supplemental Fig. S4C). There was a significant increase in the separation between *Bmi1* and *Skida1* in both *Ring1b*^−/−^ and *Ring1b^I53A/I53A^* mESCs, albeit with the latter displaying a more subtle effect consistent with that observed in the Hi-C data (Fig. 4A,B). Variable levels of increase were also observed in RING1B mutant ESCs when comparing the distance separating the PcG^−^ mid-point and either of the individual PRC1 target genes. This is consistent with the idea that PRC1 binding can impact on chromatin structure of neighboring areas with low or undetectable levels of polycomb (Fig. 4B; Supplemental Fig. S4C).

We quantified the level of interactions between PcG targets genome-wide using pileup analysis of our Hi-C data (Flyamer et al. 2020). Polycomb proteins are targeted to CpG islands (CGIs) in mammalian cells (Blackledge and Klose 2011; Deaton and Bird 2011) and so we first focused our analysis on these genomic features. We assessed distal interactions between all CGIs, CGIs lacking detectible RING1B, CGIs occupied by RING1B, and CGIs associated with both RING1B and H3K27me3 (Fig. 4C). There was no prominent enrichment of interactions between all CGIs or CGIs lacking RING1B in *Ring1b*^+/+^ mESCs, suggesting that neither the atypical base composition (high G + C and CpG) nor factors associated with these regulatory elements were sufficient to coordinate distal interactions (Fig. 4C). In contrast, a high level of enrichment was observed between CGIs bound by RING1B, and this was further enhanced at RING1B and H3K27me3 double-positive CGIs (Fig. 4C). Consistent with our previous observations, this enrichment was preserved in *Ring1b^I53A/I53A^* mESCs but lost in cells lacking RING1B (Fig. 4C).

To investigate the relationship between PRC1-mediated looping and interactions coordinated by CTCF, we performed pileup analysis of all RING1B ChIP-seq peak regions at different distance separations and compared it with the interactions of CTCF binding sites based on their orientation (Bonev et al. 2017). As expected, this revealed loop extrusion-associated structures at CTCF site intersections, including prominent loops between convergent sites (Fig. 4D**;** Rao et al. 2014; Sanborn et al. 2015; Fudenberg et al. 2016). CTCF-mediated loop intensities were highest between 100 and 400 kb, were largely undetectable at distances >1.6 Mb (Fig. 4D), and were unaffected in either of the RING1B mutant mESC lines (Supplemental Fig. S4D). In contrast, enriched contact frequencies between RING1B binding sites were detected at distances up to ∼100 Mb (Fig. 4D; Supplemental Fig. S4D). This suggests that PRC1 sites can physically associate in *cis* over very large genomic distances through a mechanism that is distinct from that driving cohesin-mediated loop extrusion. To directly test this, we investigated PRC1-mediated interactions in Hi-C data generated from mESCs bearing auxin-inducible degron-tagged CTCF (Nora et al. 2017). Associations between RING1B bound CGIs were unaffected by the loss of CTCF (“auxin”) (Supplemental Fig. S4E), confirming that the formation of PRC1-mediated interactions is mechanistically distinct from that required for loop extrusion consistent with other published observations (Rhodes et al. 2020).

To investigate whether the local abundance of PRC1 was key to defining those sites that physically interact, we stratified all RING1B peaks into quartiles based on either ChIP-seq signal strength or peak length and performed pile-up analysis on each set of regions (Fig. 4E; Supplemental Fig. S4F). We observed a much greater enrichment of interactions between the highest occupancy and longest RING1B peak regions (quartile 4 [Q4]) in both *Ring1b*^+/+^ and *Ring1b^I53A/I53A^* mESCs (Fig. 4E; Supplemental Fig. S4F). Almost no enrichment was observed in *Ring1b*^−/−^ cells even in Q4, consistent with our previous observations (Fig. 4E). This suggests that a high RING1B occupancy is critical for robust association between distal polycomb sites.

Published observations have suggested that cPRC1 complexes control polycomb-dependent 3D genome architecture, while ncPRC1s do not play a major role (Francis et al. 2004; Grau et al. 2011; Isono et al. 2013; Wani et al. 2016; Kundu et al. 2017; Plys et al. 2019; Tatavosian et al. 2019). To test this hypothesis genome-wide, we repeated the previous analysis, this time subdividing RING1B peaks based on the ratio of ChIP-seq signal between canonical and noncanonical PRC1 subunits (CBX2 vs. RYBP, respectively) (Deaton et al. 2016; Rose et al. 2016). Substantially higher interaction frequencies were observed for the relatively CBX2-enriched and RYBP-depleted peak-regions (Fig. 4F). A similar result was also obtained when we compared a different pair of canonical and noncanonical PRC1 subunits (PCGF2 and KDM2B, respectively) (Supplemental Fig. S4G; Farcas et al. 2012; Morey et al. 2015). These findings support a role for canonical PRC1 in mediating distal interactions. We noted however, that the level of RING1B enrichment was generally higher at sites relatively enriched for canonical PRC1, which, in light of our previous observations, could have potentially confounded interpretation of these results (Fig. 4F; Supplemental Fig. S4G). Therefore, we required an alternative analysis that would allow us to independently determine the relative contribution of each factor in driving distal interactions between PRC1 targets. For this, we performed individual pileups for each RING1B peak region against all other RING1B peak regions on the same chromosome. The intensity values from the central pixel of these pileups was considered as a proxy for “loopability” for each region and used to build a linear model to predict the relative contribution of different parameters on the capacity to form loops (RING1B, H3K27me3, and cPRC1 vs. ncPRC1 and peak length) (Fig. 4G). As expected, we observed a strong positive contribution of the level of RING1B binding and of peak region length. Interestingly, H3K27me3 had a relatively negative impact, confirming that PRC1 and not PRC2 (which deposits H3K27me3) is important for mediating chromatin interactions. The subunits of cPRC1 (MEL18 and CBX2) both displayed a high positive effect on loop-ability, but subunits of ncPRC1 (KDM2B and RYBP) had no or negative impact, confirming our earlier analysis and previous reports (Kundu et al. 2017). Analysis of Hi-C data from *Ring1b^I53A/I53A^* cells or of an independent *Ring1b*^+/+^ mESC data set yielded an equivalent result; however, data from *Ring1b*^−/−^ cells dramatically reduced the coefficients for all predictors (Fig. 4G; Supplemental Fig. S4H,I). Interestingly, the only positive contributors of loop formation in the absence of RING1B were PCGF2 and CBX2 (Supplemental Fig. S4H). This suggests that the very low level of interactions found in *Ring1B*^−/−^ cells is related to cPRC1, perhaps driven by complexes that instead incorporate the lowly expressed RING1A in place of RING1B.

### PRC1 mediates multivalent interactions in vitro and in vivo

**Figure 5.**
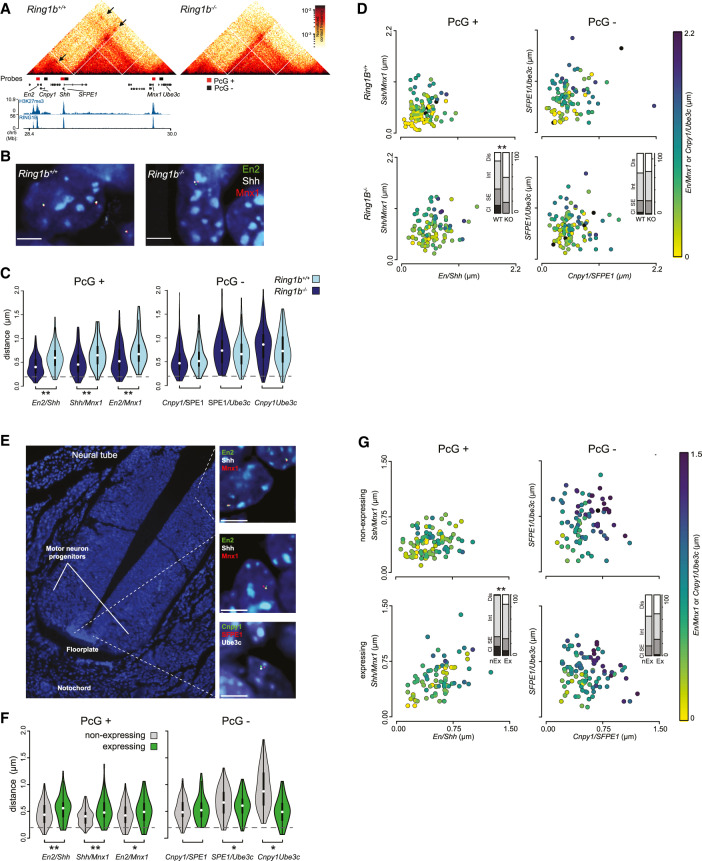
PRC1 mediates mltivalent interactions in vitro and in vivo. (*A*) Hi-C heat maps illustrating PRC1-mediated distal interactions within the *En2/Shh/Mnx1* locus (presented as for Fig. 3A; loops are highlighted with arrows). Also shown are the locations of the FISH probes used to generate *B*–*G*. (*B*) Representative 3D FISH images of *Ring1b*^+/+^ and *Ring1b*^−/−^ mESCs hybridized with the fosmid probes shown in *A* targeted to RING1B-positive and -negative sites within the locus (as illustrated in *A*; red and green bars, respectively). Scale bar, 5 μm. (*C*) Violin plots depicting the distribution of interprobe distances between each pair of fosmid probes in *Ring1b*^+/+^ and *Ring1b*^−/−^ mESCs. The significance of a shift in the distance between a given pair of probes between different cell lines is indicated. (**) *P* ≤ 0.01; Mann Whitney test. Probes separated by <0.2 μm (dashed gray line) are considered to be colocalized. (*D*) Scatter plots depicting the interprobe distances between each of the two fosmid probe pairs with the separation between the third pair indicated by the color in the color bar. Bar plots representing a categorical analysis of three-way clustering (*inset*) show the percentage of nuclei that show a clustered (Cl), single-excluded (SE), intermediate (Int), or dispersed (Dis) FISH signal. A significant shift in clustering is indicated. (**) *P* ≤ 0.01; χ^2^ test). (*E*) A 3D FISH image of a transverse section through the mouse neural tube at E10.5 with zoomed *inset* images of 3D FISH for the fosmid probes shown in *A*. (*F*,*G*) 3D FISH data presented as for *C* and *D* but for the *Shh*-expressing (floorplate) and -nonexpressing (dorsal neural tube) cell types indicated in *E*.

Domains of high PRC1 occupancy have the potential to coordinate interactions with multiple target sites simultaneously, indeed visual inspection of our Hi-C data identified examples where adjacent RING1B peaks appeared to anchor multiple overlapping loop structures (Fig. 4A; Supplemental Fig. S4A). However, as these are population average data, it was not possible to determine which and how many of these sites were able to interact simultaneously within an individual cell. To investigate this further, we focused on an ∼1.5-Mb portion of chromosome 5 that contains three genes that interact in a PRC1-dependent manner (*En2*, *Shh*, and *Mnx1*) (Fig. 5A). To investigate the 3D configuration of these loci in individual cells we performed four-color 3D FISH with probes targeting each of these genes (Fig. 5A,B). Analysis of interprobe distances showed a significant increase in the separation between each pair of target genes upon the loss of RING1B, consistent with a loss of looping (Fig. 5C; Supplemental Fig. S5A). In contrast, no changes were observed between equivalently spaced probes targeting sites that lacked detectible RING1B binding (PcG^−^) within the same region (Fig. 5A,C; Supplemental Fig. S5A). All three genes are frequently found in proximity (all pairs ≤0.35 μm) and this clustering was significantly reduced upon the loss of RING1B (Fig. 5D; Supplemental Fig. S5B). The proximity of the intervening PcG^−^ regions was not significantly altered between *Ring1b*^+/+^ and *Ring1b*^−/−^ mESCs (Fig. 5D; Supplemental Fig. S5B). These data demonstrate that PRC1 can coordinate interactions between multiple loci simultaneously.

*Shh* is transcriptionally repressed and associated with RING1B and H3K27me3 in mESCs that are derived from the inner cell mass of the blastocyst (Fig. 5A). Later in development, *Shh* becomes activated in a temporally and spatially restricted manner. To determine whether multivalent interactions were preserved in *Shh* nonexpressing cells in vivo, and if they were subsequently released upon *Shh* activation, we performed 3D-FISH for *Shh, Mnx1*, and *En2* on tissue sections from E10.5 mouse embryos, focusing on the floor plate and neural tube where *Shh* is expressed and repressed, respectively (Delile et al. 2019). Consistent with our observation in mESCs, each individual pair of genes were more spatially separated in the *Shh*-expressing cells of the floor plate when compared with the cells of the dorsal neural tube where *Shh, Mnx1*, and *En2* are repressed (Fig. 5E,F; Supplemental Fig. S5C; Delile et al. 2019; Williamson et al. 2019), and all three genes were significantly more clustered together in the dorsal neural tube than floor plate (Fig. 5F,G; Supplemental Fig. S5D). In contrast, the spatial arrangement of intervening control loci was not significantly different between the two regions and was substantially more dispersed in general than for the PRC1 target genes (Fig. 5F,G; Supplemental Fig. S5C,D). While we cannot definitively conclude that *Shh*, *En2*, and *Mnx1* are polycomb targets in the dorsal neural tube, they are enriched for H3K27me3 in whole neural tube tissue (Supplemental Fig. S5E; The ENCODE Project Consortium 2012). We conclude that *Shh* can form multivalent interactions with other repressed genes in vitro and in vivo, and these are then subsequently lost upon gene derepression and/or the loss of polycomb binding.

### Loss of distal interactions is not caused by gene activation

**Figure 6.**
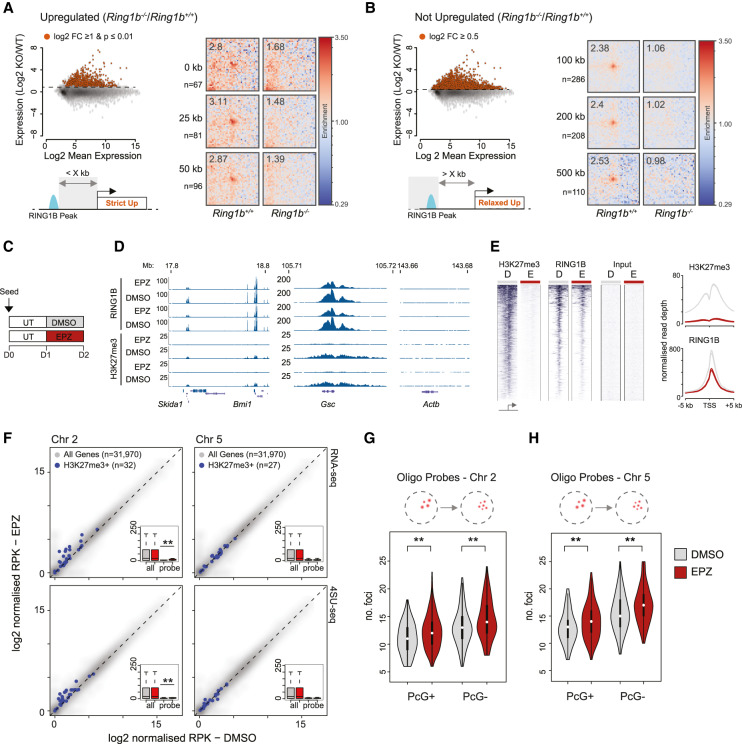
Relationship between gene up-regulation and loss of PRC1-mediated looping. (*A*) Criteria on which RING1B peaks were classified as being “up-regulated” in *Ring1B*^−/−^ versus *Ring1B*^+/+^ mESCs. (*Top left* panel) Scatter plots show the log_2_ mean gene expression versus log_2_ gene expression ratio between the two mESC lines. Red points correspond to up-regulated genes with their selection parameters noted above. (*Bottom left* panel) Cartoon schematic shows how proximity to up-regulated genes (Strict Up) was used to classify RING1B peaks for subsequent Hi-C analysis. (*Right* panel) Pileup analysis of Hi-C data illustrating PRC1 dependent distal interactions between RING1B peaks located within the indicated distance from an up-regulated gene in *Ring1B*^+/+^ and *Ring1B*^−/−^ mESCs. (n) Number of RING1B peaks used in each row. (*B*) As for *A* but representing those RING1B peaks that are distant from up-regulated genes (distant to “Relaxed Up” genes). (*C*) Scheme of EPZ6438 treatment of wild-type mESCs. (UT) serum + LIF; (DMSO) serum + LIF + DMSO; (EPZ) serum + LIF + 2.5 µM EPZ6438. (*D*) Example browser track views of RING1B and calibrated H3K27me3 ChIP-seq data from mESCs following 24 h of EPZ/DMSO treatment (two independent replicates shown). (*E*) Heat map representation (*left* panel) and summary metaplots (*right* panel) of RING1B and H3K27me3 ChIP-seq signal distribution at RefSeq gene TSS (±5 kb; enriched for their respective marks in wild-type ESCs) in EPZ/DMSO-treated mESCs. (*F*) Scatter plots showing the relative expression (RNA-seq; *top* panel) or transcription (4SU-seq; *bottom* panel) levels between mESCs treated with DMSO and EPZ for 24 h. Genes positive for H3K27me3 and located within the region covered by the oligonucleotide probes on chromosomes 2 and 5 (±100 kb; *left* and *right* plots, respectively) are highlighted in blue. *Inset* box plots summarize these values for “all” and “probe”-associated H3K27me3 positive genes in the two conditions. The significance of differential expression/transcription for genes associated with the two probes was tested using a paired Wilcoxon rank sum tests and the results indicated. (**) *P* ≤ 0.01. The number of genes representing each set are indicated in parenthesis. (*G*,*H*) Violin plots depicting the number of discrete fluorescent foci in *DMSO* (gray) and *EPZ* (red), treated mESC hybridized with either the PcG^+^ or PcG^−^ oligonucleotide probes on chromosomes 2 (*G*) and 5 (*H*). The significance of a shift in the number of discrete foci between a given pair of samples was tested using a Mann Whitney test, the results of which are indicated. (**) *P* ≤ 0.01.

Compared with wild-type cells, *Ring1b*^−/−^ mESCs have a more pronounced level of gene up-regulation than *Ring1b^I53A/I53A^* mESCs and have a substantially more altered chromatin structure. Moreover, we showed that interactions between *Shh*, *Mnx1*, and *En2* are lost upon gene activation. This raises the possibility that the loss of chromatin contacts in the absence of RING1B is simply a consequence of transcriptional activation. To investigate this, we categorized RING1B peaks as being proximal to, or distant from, genes that are up-regulated in *Ring1b*^−/−^ mESCs (Fig. 6A,B; Illingworth et al. 2015). “Up-regulated” RING1B peaks were those proximal to an up-regulated gene (0–50 kb from a gene with a strict definition of up-regulation—log2 expression ratio (Ring1b^−/−^/Ring1b^+/+^) ≥ 1 and a *P* value of ≤ 0.01; Fig. 6A). RING1B peaks were classified as not up-regulated if they were situated distant from an up-regulated gene (>100 kb from any gene with a liberal definition of up-regulation −log_2_ expression ratio; Ring1b^−/−^/Ring1b^+/+^≥0.5) (Fig. 6B). Averaged interaction strength for each category of RING1B peak was derived from pileup analysis of Hi-C data from both *Ring1b*^−/−^ and *Ring1b*^+/+^ mESCs. Looping between RING1B peaks was lost in *Ring1b*^−/−^ mESCs irrespective of whether the associated gene was up-regulated or not (Fig. 6A,B), suggesting that gene activation was not responsible for the observed loss of chromatin contacts in the absence of RING1B, and similarly that loss of interactions is not sufficient to activate genes.

To look at a more acute response to the loss of polycomb we used EPZ6438 (henceforth referred to as EPZ), a potent small molecule inhibitor of EZH1/2 that results in the passive loss of H3K27me3 (Knutson et al. 2013). We reasoned that treatment with EPZ for 24 h would reduce H3K27me3 levels sufficiently to reduce RING1B occupancy without substantially altering transcription (Illingworth et al. 2016). We seeded wild-type mESCs and cultured them for 24 h prior to treatment with either EPZ or DMSO (negative control) for a further 24 h (Fig. 6C). ChIP for H3K27me3 and RING1B followed by quantitative PCR demonstrated an almost complete loss of H3K27me3 and a substantial (∼50%) reduction in the level of RING1B at a panel of candidate genes (Supplemental Fig. S6A,B). This was confirmed genome-wide analysis by deep sequencing (ChIP-seq) (Fig. 6D,E; Supplemental Fig. S6C).

We used both RNA-seq and 4SU-seq to examine the impact of EPZ treatment on total RNA and nascent transcript levels, respectively (Rabani et al. 2011). We focused first on the *Bmi1*/*Skida1* locus (Fig. 4A) that showed a subtle but significant transcriptional up-regulation of the proximal genes (±100 kb) (Supplemental Fig. S6D,E) and a significant increase in physical separation between *Bmi1* and *Skida1* measured by 3D FISH (Supplemental Fig. S6F). At the regions covered by our oligo probes on chromosomes 2 and 5 (Fig 2A,D), there was significant low-level transcriptional up-regulation across the chromosome 2 region but not for chromosome 5 (Fig. 6F). Despite this difference, an equivalent loss of physical clustering was observed for both oligo-probe sets (Fig. 6G,H; Supplemental Fig. S6G,H). This result supports our conclusion from Hi-C analysis that transcriptional up-regulation can be associated with, but is not required for, the loss of PRC1 mediated interactions and conversely that the loss of PRC1 mediated-interactions does not necessarily lead to gene expression, at least in ES cells.

## Discussion

Transcriptional activity, protein composition, and chromatin state all play roles in specifying the spatial arrangement of the genome. Polycomb-associated facultative heterochromatin is an exemplar of this in that it mediates its own partitioning into discrete, cytologically visible nuclear polycomb bodies (Satijn et al. 1997; Saurin et al. 1998; Pirrotta and Li 2012; Isono et al. 2013; Wani et al. 2016; Plys et al. 2019; Tatavosian et al. 2019). This organization is established by PRC1 subunits that drive the formation of local compaction domains and longer-range chromatin interactions (Isono et al. 2013; Wani et al. 2016; Kundu et al. 2017). In this study we highlight the substantial contribution of PRC1-mediated interactions in controlling overall nuclear architecture and explore its connection to gene activity.

### PRC1 and nuclear architecture

**Figure 7.**
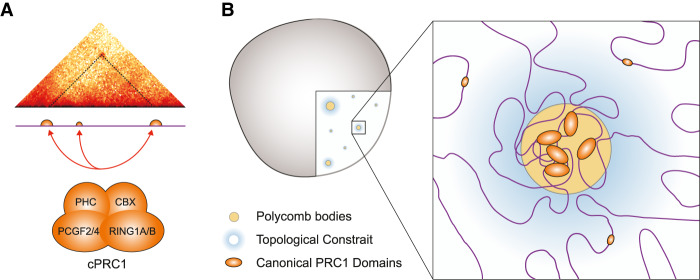
Canonical PRC1 influences gross nuclear organization. (*A*) PcG mediated interactions occur preferentially between sites with the most pronounced and extended domains of canonical PRC1 occupancy. (*B*) We propose that PRC1-bound loci cluster together in the nucleus to form discrete polycomb bodies. This clustering, coupled with the polymeric nature of chromatin, imposes topological constraint on intervening non-PcG-associated portion of the genome.

Using FISH, we demonstrated that cells lacking RING1B have substantially larger nuclei and display reduced clustering of polycomb target loci. This suggests that while occupying <1% of the linear genome, RING1B has a marked impact on global nuclear organization. By Hi-C, we observed that only those loci with the most pronounced binding of canonical PRC1, and not noncanonical PRC1, produced detectibly enriched interactions (Fig. 4E–G; Supplemental Fig. S4F–I). By comparing chromatin contacts in the presence and absence of RING1B or CTCF, we conclude that PRC1-mediated interactions are independent of CTCF, a finding consistent with published observations (Rhodes et al. 2020). We show that PRC1 anchors chromosomal contacts at genomic distances of up to 100 times longer than those of CTCF, suggesting that this architecture is distinct from and independent of TADs, both in terms of mechanism and scale. We also show that PRC1-mediated chromatin interactions can be multivalent (Fig. 5). This is consistent with the observation that canonical PRC1 can drive liquid–liquid phase separation; a biophysical process that depends on weak multivalent interactions and can lead to nuclear compartmentalization and the segregation of both active and inactive chromatin states (Hnisz et al. 2017; Larson and Narlikar 2018; Plys et al. 2019; Tatavosian et al. 2019). The polymeric nature of chromatin means that clustering of PcG sites will impose topological constraint on the intervening nonpolycomb-associated portion of the genome as we observed here (Fig. 7).

### PRC1 functionality and gene expression

PRC1 modulates both the structure and modification state of chromatin; functions grossly ascribed to canonical and noncanonical PRC1, respectively (Grau et al. 2011; Isono et al. 2013; Blackledge et al. 2014,^2020^; Rose et al. 2016; Kundu et al. 2017; Plys et al. 2019; Tatavosian et al. 2019). What then is the relative contribution of these functions to gene repression? Here we show that a hypomorphic form of RING1B, with substantially impaired catalytic activity largely preserves normal chromosomal architecture (Figs. 2–4; Supplemental Figs. S2–S4). As this mutation yields only modest gene expression changes and phenotypic consequences during embryonic development (Illingworth et al. 2015; Kundu et al. 2017; Cohen et al. 2018), this suggests that modulating chromosomal architecture and not H2AK119Ub is the primary repressive activity. However, induced disruption of PCGF2 and PCGF4, which cripples canonical PRC1 specifically, leads to minimal gene up-regulation (Fursova et al. 2019). Furthermore, induced loss of the variant PRC1 complexes that are responsible for H2AK119ub deposition or the complete disruption of RING1B E3 ubiquitin ligase activity leads to a substantial up-regulation of gene expression in mESCs (Fursova et al. 2019; Blackledge et al. 2020; Tamburri et al. 2020). This suggests that low levels of H2AK119Ub are sufficient for PRC1-mediated gene repression and chromatin folding, possibly by contributing to efficient polycomb recruitment (Blackledge et al. 2014; Cooper et al. 2014).

Mice bearing mutations in PRC1 subunits with structural functions display homeotic transformations indicative of *Hox* gene misregulation (Isono et al. 2013; Lau et al. 2017). Although, subtle in comparison with the gastrulation arrest observed in *Ring1b*^−/−^ embryos, this observation posits two nonmutually exclusive scenarios. First, that a small subset of key developmental regulators is repressed by a PRC1-mediated refractory chromatin configuration. Indeed, in the absence of E3 ubiquitin ligase activity or variant PRC1 complexes, a small subset of genes (100–200), including *Hox* genes, remains repressed (Fursova et al. 2019; Blackledge et al. 2020). A comparison between these genes and our Hi-C data showed that over half form RING1B-dependent distal interactions in mESCs (data not shown). A second possibility is that PRC1-mediated interactions in mESCs establish an architectural configuration that facilitates subsequent gene activation. Indeed, it has been shown that polycomb-dependent interactions can connect repressed gene promoters and their poised enhancers, and that this is critical for correct gene activation upon neural induction (Kondo et al. 2014; Vieux-Rochas et al. 2015).

### Concluding remarks

In this study we show that PRC1 substantially impacts on nuclear organization and provide the first demonstrable example of expression state-dependent multivalent interactions between polycomb target sites during embryonic development. While we show that it is possible to uncouple PRC1-mediated interactions from gene repression, many expression changes do accompany loss of chromatin contacts. However, no study has directly demonstrated a causal link between PRC1's architectural function and gene repression in mammalian cells (Bantignies et al. 2003, 2011). Furthermore, the stoichiometry of CBX subunits alters dramatically during differentiation favoring, in the case of neural progenitor cells, the capacity to compact nucleosomal templates (Grau et al. 2011; Kloet et al. 2016). Further work is required to fully appreciate the role of PRC1-mediated interactions in the repression and/or timely activation of gene expression programs during embryonic development.

## Materials and methods

### Tissue culture

Feeder-free mouse embryonic stem cells (mESCs) including E14tg2A (129/Ola; *Ring1B*^+/+^) and the derivative lines (*Ring1B*^I53A/I53A^ and *Ring1B*^−/−^) (Illingworth et al. 2015) were cultured on 0.1% gelatin-coated (Sigma G1890) Corning flasks in GMEM BHK-21 (Gibco 21710-025) supplemented with 10% fetal calf serum (FCS; Sigma F-7524), 1000 U/mL LIF, nonessential amino acids (Gibco 11140-035), sodium pyruvate (Gibco 11360-039), 50 μM 2-β-mercaptoethanol (Gibco 31350-010), and L-glutamine. For passaging, 60%–90% confluent ESC culture flasks were washed with PBS, incubated for 2–3 min at room temperature in 0.05% (v/v) trypsin (Gibco 25300-054), and tapped to release. Trypsin was inactivated by adding 9 vol of ESC medium, and this mixture was repeatedly pipetted to obtain a single-cell suspension. ESCs were centrifuged, resuspended in ESC medium, and replated onto gelatin-coated flasks at a density of ∼4 × 10^4^ cells/cm^2^ (determined using a hemocytometer; Neubauer). For short-term EZH1/2 inhibition experiments, ESCs were plated in standard medium at 4 × 10^4^ cells/cm^2^ and cultured for 24 h. The medium was then replaced with medium supplemented with either EPZ-6438 (BioVision 2383-5; reconstituted in DMSO) at a final concentration of 2.5 µM or DMSO, and cultured for a further 24 h prior to harvesting or analysis.

Feeder-dependent mESCs (*Ring1B*^+/+^ and *Ring1B*^−/−^) (Leeb and Wutz 2007) were plated on a layer of mitomycin C-inactivated primary embryonic fibroblasts (PEFs; derived from E12.5 mouse embryos), and grown in DMEM (Gibco 41965-039) supplemented with 15% fetal calf serum, 1000 U/mL LIF, nonessential amino acids (Sigma M7145), sodium pyruvate (Sigma S8636), 2-β-mercaptoethanol (Gibco 31350-010), and L-glutamine. Passaging was performed as above.

For 3D FISH, mESCs were seeded onto gelatin-coated Superfrost Plus microscope slides (Thermo Fisher Scientific J1800AMNT). Feeder-free ESCs (2.5 × 10^5^) were seeded onto slides and cultured for 48 h prior to processing. For feeder-dependent cells, PEFs were removed through two consecutive rounds of preplating (twice for 30 min in LIF-containing medium at 37°C) before plating 1 × 10^6^ mESCs per slide. After ∼6 h of incubation at 37°C, cells were sufficiently adherent to process for FISH.

All centrifugation steps with live cells were performed at 330*g* for 3 min at room temperature. All ESC lines used in this study were routinely tested for mycoplasma.

### DNA 3D fluorescent in situ hybridization (DNA-FISH)

#### Fixation

Mouse embryonic tissue sections were prepared as previously described (Morey et al. 2007). Mouse ESCs grown on slides were fixed in 4% PFA, permeabilized in PBS/0.5% Triton X, dried, and then stored at −80°C prior to hybridization. Slides were incubated in 100 μg/mL RNase A in 2× SSC for 1 h at 37°C, washed briefly in 2× SSC, passed through an alcohol series, and air-dried. Slides were incubated for 5 min at 70°C, denatured in 70% formamide/2× SSC (pH 7.5) for 40 min at 80°C, cooled in 70% ethanol for 2 min on ice, and dehydrated by immersion in 90% ethanol for 2 min and 100% ethanol for 2 min prior to air drying.

#### Hybridization and analysis

Eight-hundred nanograms of each fluorescently labeled oligonucleotide probe pool (2 μL; MyTags or Roche Probes) was added to 26 μL of hybridization mix (50% formamide, 2× SSC, 1% Tween20, 10% dextran sulfate), denatured for 5 min at 70°C, and then snap-chilled on ice.

One microgram of fosmid DNA was labeled by nick translation to incorporate green-dUTP (Enzo Lifesciences), Alexa fluor 594-dUTP (Invitrogen) or aminoallyl-dUTP-ATTO-647N (Jena Biosciences). One-hundred nanograms of fosmid, 6 μL of Cot1 DNA, and 5 μg of sonicated salmon sperm DNA were dried in a spin-vac and then reconstituted in 30 μL of hybridization mix. Probes were then denatured for 5 min at 80°C and reannealed for 15 min at 37°C.

Fosmid and oligonucleotide probes were hybridized to slides under a sealed coverslip overnight at 37°C. Slides were washed the next day four times for 3 min in 2× SSC at 45°C and four times for 3 min in 0.1× SSC at 60°C, stained with 4,6-diaminidino-2-phenylidole (DAPI) at 50 ng/mL, mounted in VectaShield (Vector Laboratories), and sealed with nail varnish.

Epifluorescent images were acquired using a Photometrics Coolsnap HQ2 CCD camera and a Zeiss AxioImager A1 fluorescence microscope with a plan apochromat 100× 1.4NA objective, a Lumen 200-W metal halide light source (Prior Scientific Instruments) and Chroma 89014ET single-excitation and emission filters (three-color FISH) or Chroma 89000ET single-excitation and emission filters (four-color FISH) (Chroma Technology Corp.) with the excitation and emission filters installed in Prior motorized filter wheels. A piezoelectrically driven objective mount (PIFOC model P-721, Physik Instrumente GmbH & Co.) was used to control movement in the z dimension. Hardware control, image capture, and analysis were performed using Volocity (Perkinelmer, Inc.) or Nis elements (Nikon)

Images were deconvolved using a calculated point spread function with the constrained iterative algorithm of Volocity.

Image analysis was carried out using the quantitation module. To ensure unbiased scoring, FISH slides were ascribed nondescriptive identifiers allowing image processing and visual scoring to be performed blind to genotype or treatment.

Additional information relating to all FISH probes used in this study is outlined in Supplemental Table S1.

### Calibrated ChIP sequencing (ChIP-seq)

Trypsinized mESCs (20 × 10^6^) were washed twice in PBS. Cells were resuspended in 250 µL of PBS and fixed by the addition of an equal volume of PBS containing 2% methanol-free formaldehyde (Thermo Scientific Pierce PN28906; final concentration of 1%) and incubated for 10 min at room temperature. Fixation was stopped by 5-min incubation with 125 mM of glycine at room temperature. Fixed cells were washed in PBS and combined at this stage with 1.3 × 10^6^ formaldehyde-fixed S2 cells (*Drosophila melanogaster* cells; for downstream calibration of ChIP-seq data). All buffers were supplemented with 1 mM DTT and 1× Protease inhibitors (Roche 11836170001) just prior to use. Cell pellets were resuspended in lysis buffer (50 mM Tris-HCl at pH 8.1, 10 mM EDTA, 20% SDS) and incubated for 10 min at 4°C. Lysates were diluted 1:10 in ChIP dilution buffer (1% Triton X-100, 2 mM EDTA, 150 mM NaCl, 20 mM, Tris-HCl at pH 8.1) and sonicated, first with a single 30-sec pulse with a probe sonicator (Labtech Soniprep 150) on ice followed by a further 45 cycles using a cooled Bioruptor (Diagenode; 1-min cycles of 30 sec on/30 sec off on “high” setting at 4°C). The sonicated extract was precleared by centrifugation at 16,000*g* for 10 min at 4°C. The supernatant was transferred to a fresh tube and supplemented with BSA to a final concentration of 25 mg/mL. A sample of the chromatin was retained as an input reference. Antibodies were precoupled to protein A Dynabeads (Life Technologies 10001D) at a ratio of 1 mg antibody per 30 mL of Dynabead suspension by rotation for 1 h at 4°C. Cell equivalents (12 × 10^6^ and 6 × 10^6^) of lysate were added to 7.5 µg of anti-Ring1B (Cell Signaling D22F2) or 5 µg of anti-H3K27me3 (Cell Signaling C36B11), respectively, and incubated for 6 h on a rotating wheel at 4°C. Following incubation, bead-associated immune complexes were washed sequentially with ChIP dilution buffer, wash buffer A, and wash buffer B, each for 10 min at 4°C on a rotating wheel, followed by two washes in TE buffer at room temperature (wash buffer A: 1% Triton X-100, 0.1% sodium-deoxycolate, 0.1% SDS, 1mM EDTA, 500 mM NaCl, 50 mM HEPES at pH 7.9; wash buffer B: 0.5% NP40, 0.5% sodium-deoxycolate, 1 mM EDTA, 250 mM LiCl, 20 mM Tris-HCl at pH 8.1). Chromatin was released by incubating the beads in 100 µL of elution buffer (0.1 M NaHCO_3_, 1% SDS) for 15 min at 37°C, followed by the addition of 50 µg of RNase A and 6 µL of 2 M Tris (pH 6.8) and incubation for 2 h at 65°C and finally by the addition of 50 µg of proteinase K and incubation for 8 h at 65°C to degrade proteins and reverse the cross-links. Dynabeads were removed using a magnetic rack and the chromatin purified using PCR purification columns (Qiagen) according to the manufacturer's instructions.

Libraries were constructed using the NEBNext Ultra II DNA library preparation kit for Illumina according to the manufacturer's instructions (NEB E7645S). Library PCRs were supplemented with 2× SYBR dye (Sigma S9430) so that amplification could be monitored by quantitative PCR on a Roche LightCycler 480. To allow for sample multiplexing, PCRs were performed using index primers (NEBNext multiplex oligos for Illumina, set 1, E7335) and amplified to linear phase. Size selection purifications following the ligation and amplification PCR steps were performed with 1× and 0.9× reaction volumes of Agencourt AMPure XP beads (Beckman Coulter A63880). Purified libraries were combined as a 12-sample equimolar pool containing the indexes 1–12 and sequenced on an Illumina NextSeq on a single high-output flow cell (single-end 75-bp reads).

### 4SU sequencing (4SU-seq)

4SU-seq was performed essentially as described previously (Rabani et al. 2011). Briefly, 4-thiouridine (4SU; Sigma T4509) was added to ESCs in culture to a final concentration of 500 µM and incubated for 20 min at 37°C. Cells were harvested by trypsinization and washed twice with PBS at room temperature. Total RNA was isolated from 7 × 10^6^ cells using Trizol according to the manufacturer's instructions (Invitrogen 15596026). Following precipitation, purified RNA was resuspended in 100 μL of RNase-free water and DNase-treated using the Turbo DNA-free kit according to the manufacturer's instructions (Invitrogen AM1907M). Residual inactivation beads were removed by spinning the RNA sample through a QIAshredder column at 1000*g* for 1 min (Qiagen). Two micrograms of total RNA input was retained for each sample and 30 μg was incubated for 1.5 h at room temperature with 60 μg of Biotin-HPDP (Pierce 21341; reconstituted in dimethylformamide at 1 mg/mL) in 1× biotinylation buffer (10 mM Tris at pH 7.4, 1 mM EDTA) to a total volume of 300 μL. Uncoupled biotin was removed through two consecutive rounds of 1:1 (v/v) chloroform extraction, followed by isopropanol/NaCl precipitation. RNA was resuspended in 100 μL of RNase-free water and mixed 1:1 (w/w) with µMacs Streptavidin beads (Miltenyi 130-074-101) and incubated for 15 min at room temperature with rotation. The RNA/bead mixture was applied to a µMacs column following pre-equilibration with wash buffer (100 mM Tris at pH 7.5, 10 mM EDTA, 1 M NaCl, 0.1% Tween20). The captured beads were then washed three times with 900 µL of 65°C wash buffer and three times with 900 µL of room temperature wash buffer. RNA was then eluted from the column by adding two consecutive rounds of 100 mM DTT. The eluate was added to 700 µL of buffer RLT (RNeasy MinElute cleanup kit; Qiagen 74204) and then purified according to the manufacturer's instructions. Prior to library preparation, ribosomal RNA was depleted from both the total and purified nascent RNA using the low-input RiboMinus eukaryote system v2 kit according to the manufacturer's instructions (Ambion A15027).

Libraries were constructed using the NEBNext Ultra II directional RNA library preparation kit for Illumina according to the protocol for ribosome-depleted RNA and with an 11-min RNA fragmentation step (NEB E7760). Library PCRs were supplemented with 2× SYBR dye (Sigma S9430) so that amplification could be monitored by quantitative PCR on a Roche LightCycler 480. To allow for sample multiplexing, PCRs were performed using index primers (NEBNext multiplex oligos for Illumina, set 1, E7335) and amplified to linear phase. Size selection purifications following the ligation and amplification PCR steps were performed with 1× and 0.9× reaction volumes of Agencourt AMPure XP beads (Beckman Coulter A63880). Purified libraries were combined as an eight-sample equimolar pool containing the indexes 5–12 and sequenced on an Illumina NextSeq on a single high-output flow cell (paired-end 75-bp reads).

### ChIP-seq analysis

Published ChIP-seq data from mESCs (GEO accessions: RING1B, GSM1713906-7; H3K27me3, GSM1713910-11; MEL18, GSM1657387; CBX2, GSM2080677; KDM2B, GSM1272789-91; and RYBP, GSM2192980-82) (Blackledge et al. 2014; Illingworth et al. 2015; Morey et al. 2015; Deaton et al. 2016; Rose et al. 2016) was retrieved from the Short Read Archive (SRA). SRA files was converted to Fastq using fastq-dump from the SRA toolkit.

#### Mapping and processing

ChIP-seq data were mapped to the mouse genome (mm9 build) using bowtie2 with the –local –threads 3 -S options to generate SAM files. Using the HOMER package, SAM files were converted into tag directories and multimapping reads were removed using makeTagDirectory -unique -fragLength 150. Mapped regions that, due to fragment processing, extended beyond the end of the chromosomes were removed using removeOutOfBoundsReads.pl with chromosome lengths for mm9. Replicate data, where appropriate, were combined at this stage. Genome browser files (.bw) were generated using makeUCSCfile with the -bigWig -fsize 1e20 -norm 10e7 -color 25,50,200 options. H3K27me3 genome browser files were normalized instead to a calibrator value set to maintain the relative contribution of *Drosophila* spike-in reads between the input and immunoprecipitated samples.

#### Signal quantitation

For ChIP quantitation, published RING1B peaks (Illingworth et al. 2015) separated by <5000 bp were merged using bedtools mergeBed function with -d 5000. HOMER was then used to quantify read coverage across these merged regions. For linear modeling (see “Hi-C Data Analysis” below) simple read coverage was determined using a*nnotatePeaks.pl* with the following parameters -size “given” -noann -nogene -len 0 -strand both -norm 10e7. Window files centered on RING1B peaks (±5 kb) used to make heat maps were generated using annotatePeaks.pl **-**size 10000 -hist 200 -ghist -nogene -strand both -norm 10e7 (calibrated normalization for H3K27me3 chIP-seq data was performed as outlined above). For the comparison of HiC contact frequency with RING1B/H3K27me3 occupancy, ChIP signal was quantified across the whole mouse genome in 25-kb abutting windows using annotatePeaks.pl with the same parameters as simple RING1B peak quantitation outlined above. Where appropriate all quantifications were expressed as reads per kilobase per million mapped reads (RPKM).

#### CGI analysis

The coordinates of biochemically defined mouse CGIs (mm9) (Illingworth et al. 2010) were intersected with published peaks of RING1B and H3K27me3 (Illingworth et al. 2015) using the intersect function of Bedtools with the following paramaters -wa -u –a.

### 4SU-seq and RNA-seq analysis

#### Mapping and processing

For each demultiplexed sample, multiple raw Fastq files were merged (individually for reads 1 and 2) and then aligned to the mouse genome (mm9) using Bowtie2 v2.2.6 for paired-end sequence data (options: –local –threads 3) to generate .SAM files. Aligned read data were processed using HOMER v4.8. SAM files were converted into tag directories using “makeTagDirectory” with the following parameters: -format sam -flip –sspe. Genomic intervals that extended beyond the end of the chromosomes were removed using “removeOutOfBoundsReads.pl.” Strand-specific browser track files (bigwig format; “.bigWig”) for each replicate were generated using “makeUCSCfile” with the following parameters: -fsize 1e20 -strand + (or −) -norm 1e8.

#### Signal quantitation

HOMER was used to quantify 4sU/RNA-seq read coverage across all RefSeq genes (mm9). Coverage was determined using annotatePeaks.pl with the parameters: -size “given” -noann -nogene -len 0 -strand both -norm 0. All expression values were then converted into reads per kilobase per million mapped reads (RPKM) using R (https://www.r-project.org).

### In situ Hi-C

We performed in situ Hi-C largely in accordance with Rao et al. (2014) with minor modifications, same as in McLaughlin et al. (2019). Briefly, the modifications included digestion using DpnII instead of MboI (in the DpnII buffer, with previous washes in NEBuffer 3), no phenol-chlorophorm extraction after decross-linking with buffer exchange using Amicon filter units (30 kD, 500 µL), sonication using a probe-based sonicator to achieve fragment length distribution of ∼200–700 bp followed by concentration on Amicon filter units, indexing using barcoded primers instead of adaptors, and size selection of the final amplified library through gel extraction instead of AMPure beads. Final Hi-C libraries were test-sequenced at the Wellcome Trust Clinical Research Facility (Edinburgh) on NextSeq550 PE75, and selected high-quality (by *cis*/*trans* ratio and consistent P_c_[s] curve) libraries were deep-sequenced at BGI on HiSeq4000 PE 100. We used two (I53A and KO) and four (WT) replicate libraries per condition with a total of ∼0.85 billion to 1.18 billion reads.

### Hi-C data analysis

Hi-C data were analyzed using the distiller pipeline (https://github.com/mirnylab/distiller-nf) on Eddie3 cluster of the University of Edinburgh. Mapping was performed to the mm9 genome assembly, and PCR and optical duplicates were removed with the max_mismatch_bp: 0 option. Data were filtered to remove reads with mapq < 30, binned to generate multiresolution. Cooler files and balanced using default parameters. The same analysis was performed with deep Hi-C data from ES cells (Bonev et al. 2017).

For insulation analysis, we used Cooltools Diamond-insulation with 25-kb resolution data and 1-Mb window size. For eigenvector analysis, we used Cooltools call compartments with 200-kb resolution data and reference track of GC content. For both analyses, we then clustered the genome-wide insulation profile or eigenvector using Seaborn.Clustermap with default algorithm setting.

Pileup analysis was performed using coolpup.py (Flyamer et al. 2020). All distal and nonrescaled local pileups used chromosome-wide expected normalization; local rescaled pileups were normalized to randomly shifted control regions (10 per region of interest). Unless specified, we did not consider regions closer than 100 kb. Pileups investigating enrichment at different distance scales used coolpup.py's ‐‐mindist and ‐‐maxdist options to specify distance ranges. Local rescaled pileups were created with ‐‐rescale_size 75 ‐‐rescale_pad 2 ‐‐minsize 10000 options.

Loop-ability was calculated using –by_window of coolpup.py. We took the enrichment1 values, corresponding to the observed/expected contacts in the central pixel of the pileups, and did not perform any filtering based on coefficient of variation. This table was merged with information about level of binding/occupancy of different factors determined by ChIP-seq. We only considered regions with enrichment1 > 0 in all three data sets, and <1000 reads of RING1B (since regions with higher coverage represented technical artifacts). We used scikit-learn to perform linear modeling using a subclass of linear_model.LinearRegression that also calculates *P*-values for each predictor (https://stackoverflow.com/a/27975633/1304161). We used properties of merged RING1B ChIP-seq peaks (see above): number of reads from ChIP-seq of H3K27me3, RING1B, MEL18, CBX2, KDM2B, and RYBP, and merged peak length. All predictor values were normalized using preprocessing.StandardScaler() method of scikit-learn. We then used the values of coefficients for each predictor to compare their relative importance for looping interactions between RING1B peaks.

Local compaction analysis was performed as described in McLaughlin et al. (2019). Briefly, total number of normalized observed/expected contacts in 25-kb windows was calculated, excluding the two first diagonals and any regions containing filtered out bins. This was then compared with the total number of ChIP-seq reads of RING1B or H3K27me3 from these regions.

#### Expression versus distal interactions

To investigate the contribution of the loss of RING1B protein versus gene derepression on distal interactions we compared our Hi-C data with published gene expression data for *Ring1b*^+/+^ and *Ring1b*^−/−^ mESCs (Illingworth et al. 2015). Refseq genes were classified as having either “strict” up-regulation (log_2_ fold change ≥1 and an adjusted *P*-value of ≤0.01) or “relaxed” up-regulation (log_2_ fold change ≥0.5) in *Ring1b*^−/−^ versus *Ring1b*^+/+^ mESCs. Hi-C pileups were generated for RING1B peaks with the highest ChIP-seq signal (upper quartile [Q4]) either for peaks associated with up-regulated gene (proximal to “strict” genes) or not associated with up-regulated genes (distant from “relaxed” genes). A range of gene-to-peak distances were assessed.

#### Statistical testing of differential interaction frequencies

Observed/expected signal ratios for individual genomic regions of interest (ROI) were extracted and used to determine the average level of interaction enrichment for that region for each Hi-C data set. Matched values from 1000 random regions of the same shape and size were determined (matched for chromosome and distance from the matrix diagonal). These permuted values were subsequently used to estimate the mean and standard deviation of the distribution of all regions for the chromosome. Following log transformation these values were used to generate *Z* score for the region of interest. The mean was subtracted from the observed value for the ROI and divided by its standard deviation and subsequently converted into a *P*-value for ease of interpretation [as 1-scipy.special.ndtr(zscore)].

### Data availability

All sequencing data was submitted to the GEO repository under accession numbers GSE134826 (Hi-C) and GSE140894 (4sU/RNA-seq and ChIP-seq).

## Supplementary Material

Supplemental Material
