# Supplementary material for: A central role for canonical PRC1 in shaping the 3D nuclear landscape
Source: Genes Dev. 2020 Jul 1;34(13-14):931–49. doi: 10.1101/gad.336487.120 (PMC7328521; doi:10.1101/gad.336487.120)
Supplement: Supplemental Material [file supp_34_13-14_931__index.html]

A central role for canonical PRC1 in shaping the 3D nuclear landscape — Supplemental Material 

# A central role for canonical PRC1 in shaping the 3D nuclear landscape

## Supplemental Material

- Supplemental\_Fig\_1.pdf
- Supplemental\_Fig\_2.pdf
- Supplemental\_Fig\_3.pdf
- Supplemental\_Fig\_4.pdf
- Supplemental\_Fig\_5.pdf
- Supplemental\_Fig\_6.pdf
- Supplemental\_Table1.xlsx
