# Supplementary material for: A central role for canonical PRC1 in shaping the 3D nuclear landscape
Source: Genes Dev. 2020 Jul 1;34(13-14):931–49. doi: 10.1101/gad.336487.120 (PMC7328521; doi:10.1101/gad.336487.120)
Supplement: Supplemental Material [file supp_gad.336487.120_Supplemental_Fig_1.pdf]

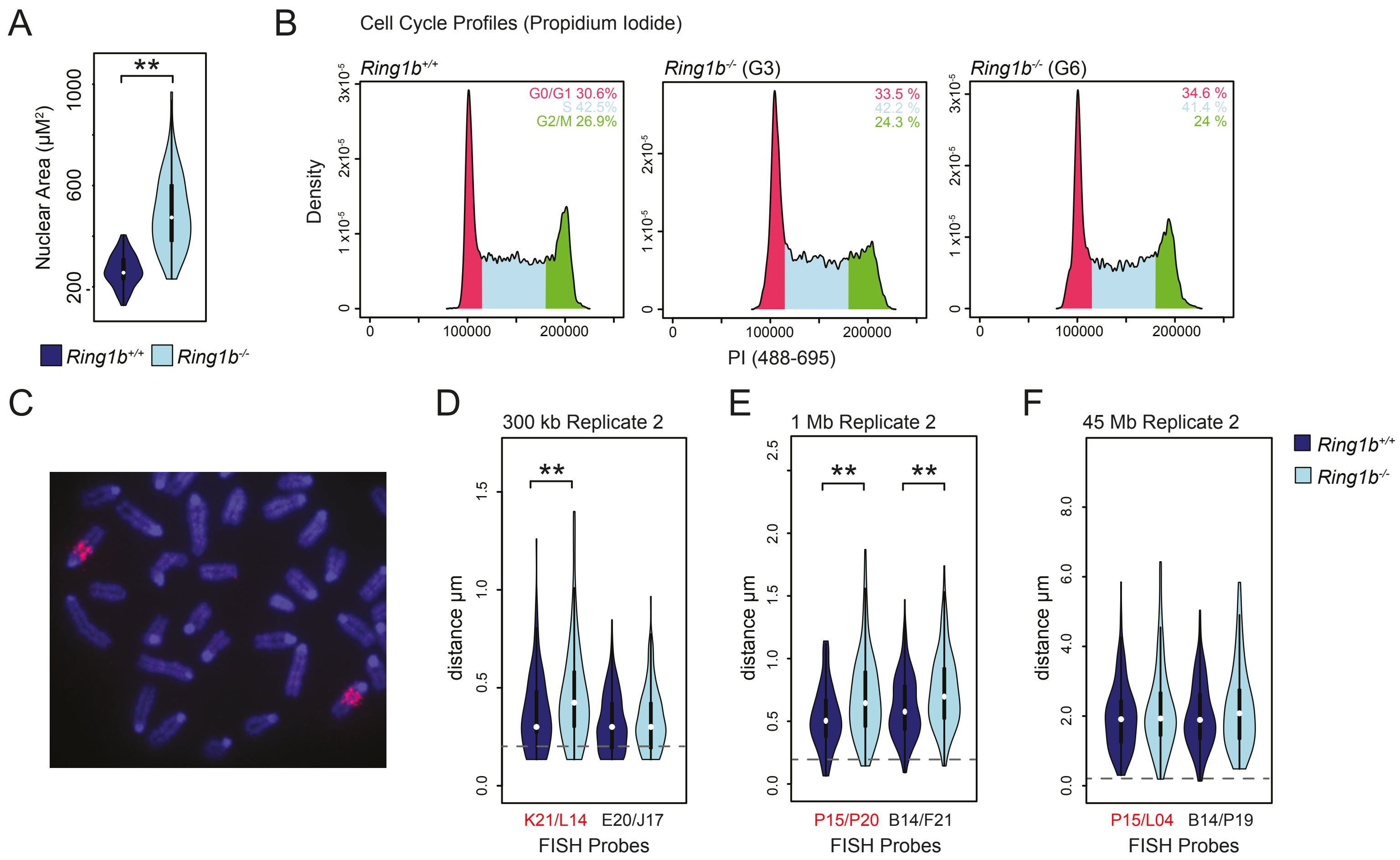

Supplemental Fig. 1. Cytological Analysis of Ring1b+/+ vs. Ring1b-/- mESCs. (A) Violin plot depicting the nuclear area of *Ring1b+/+* and *Ring1b-/-* mESCs determined by DAPI staining of 2D nuclear preparations (independent replicate experiment to that shown in **Fig. 1A**). A significant shift in nuclear area is indicated (**p = 6.94×10^{-19}; as determined by a Mann-Whitney test). **(B)** Histogram profiles of fluorescence-activated cell sorting (FACS) data from propidium iodide stained *Ring1b+/+* and *Ring1b-/-* mESCs (two independent *Ring1b-/-* clones are shown - G3 and G6). The relative distribution of cells in G0/G1, S and G2/M are indicated in parenthesis (red, blue and green respectively). **(C)** Representative FISH image of the chromosome 6 polycomb positive oligonucleotide probe signal on metaphase chromosomes from wildtype mESCs. **(D-F)** Violin plots of inter-probe distances for the indicated fosmids (locations shown in **Fig. 1C**) in *Ring1b+/+* and *Ring1b-/-* mESCs (independent replicate experiment to that shown in **Fig. 1G-I**). Probes separated by < 0.2 μm (dashed grey line) are considered to be co-localised. A significant shift in inter-probe distance between *Ring1b+/+* and *Ring1b-/-* mESCs is indicated (**p = 2.38×10^{-3} **(G)** and **p = 2.09×10^{-5} and **7.88×10^{-5} **(H)**; as determined by Mann Whitney test).
