# Supplementary material for: A central role for canonical PRC1 in shaping the 3D nuclear landscape
Source: Genes Dev. 2020 Jul 1;34(13-14):931–49. doi: 10.1101/gad.336487.120 (PMC7328521; doi:10.1101/gad.336487.120)
Supplement: Supplemental Material [file supp_gad.336487.120_Supplemental_Fig_2.pdf]

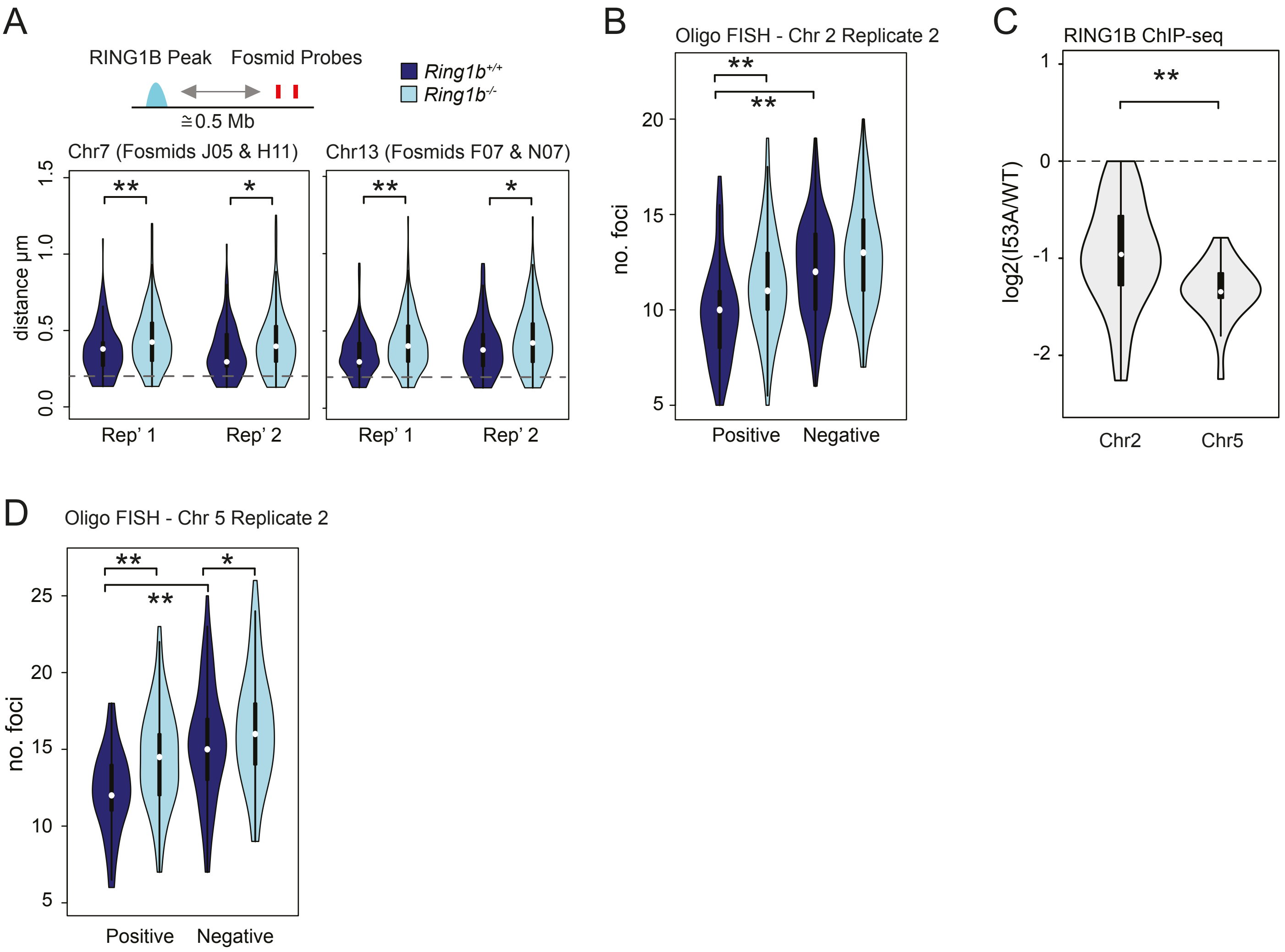

Supplemental Fig. 2. Loss of PRC1 Reduces Nuclear Clustering at Sites Lacking RING1B. (A) Violin plots showing the distribution of inter-probe distances determined by 3D FISH of *Ring1b*$^{+/+}$ and *Ring1b*$^{-/-}$ mESCs hybridised with fosmid probes situated at non-RING1B sites on chromosome 7 and 13 (left and right panels respectively; fosmids are located approximately 0.5 Mb from the closest RING1B peak). A significant shift in inter-probe distance, as determined by a Mann Whitney test, is indicated (**p = 7.79×10^{-5}, *p = 1.53×10^{-2}, **p = 6.54×10^{-4} and *p = 1.58×10^{-2} from left to right). Genomic locations are given for the mm9 genome build. Each plot shows the result of two independent replicates. Probes separated by less than 0.2 µm (dashed grey line) are considered to be co-localised. **(B)** Violin plots depicting the number of discrete fluorescent foci in *Ring1b*$^{+/+}$ and *Ring1b*$^{-/-}$ mESCs hybridised with either the polycomb positive or negative oligonucleotide probes on chromosome 2 (second independent replicate to that shown in **Fig. 2C**). The significance of a shift in the number of discrete foci between a given pair of samples was tested using a Mann Whitney test, the results of which are indicated (**p ≤ 0.01). **(C)** Violin plot showing the log2 ratio (*Ring1b*$^{I53A/I53A}$ vs. *Ring1b*$^{+/+}$) of RING1B ChIP-seq signal across all RING1B peaks within the chromosome 2 (42,398-93,905 Mb) and chromosome 5 (42.40-93.91 Mb) regions representing the PcG+ positive and a second proposed PcG+ oligonucleotide probe respectively (± 100 kb). A significantly greater reduction in RING1B levels across the chromosome 5 region was observed (**p = 0.00114; Mann Whitney test). **(D)** Violin plots depicting the number of discrete fluorescent foci in *Ring1b*$^{+/+}$ and *Ring1b*$^{-/-}$ mESCs hybridised with either the polycomb positive or negative oligonucleotide probes on chromosome 5 (second independent replicate to that shown in **Fig. 2F**). The significance of a shift in the number of discrete foci between a given pair of samples is indicated (*p ≤ 0.05 and **p ≤ 0.01; Mann Whitney test).
