# Supplementary material for: A central role for canonical PRC1 in shaping the 3D nuclear landscape
Source: Genes Dev. 2020 Jul 1;34(13-14):931–49. doi: 10.1101/gad.336487.120 (PMC7328521; doi:10.1101/gad.336487.120)
Supplement: Supplemental Material [file supp_gad.336487.120_Supplemental_Fig_3.pdf]

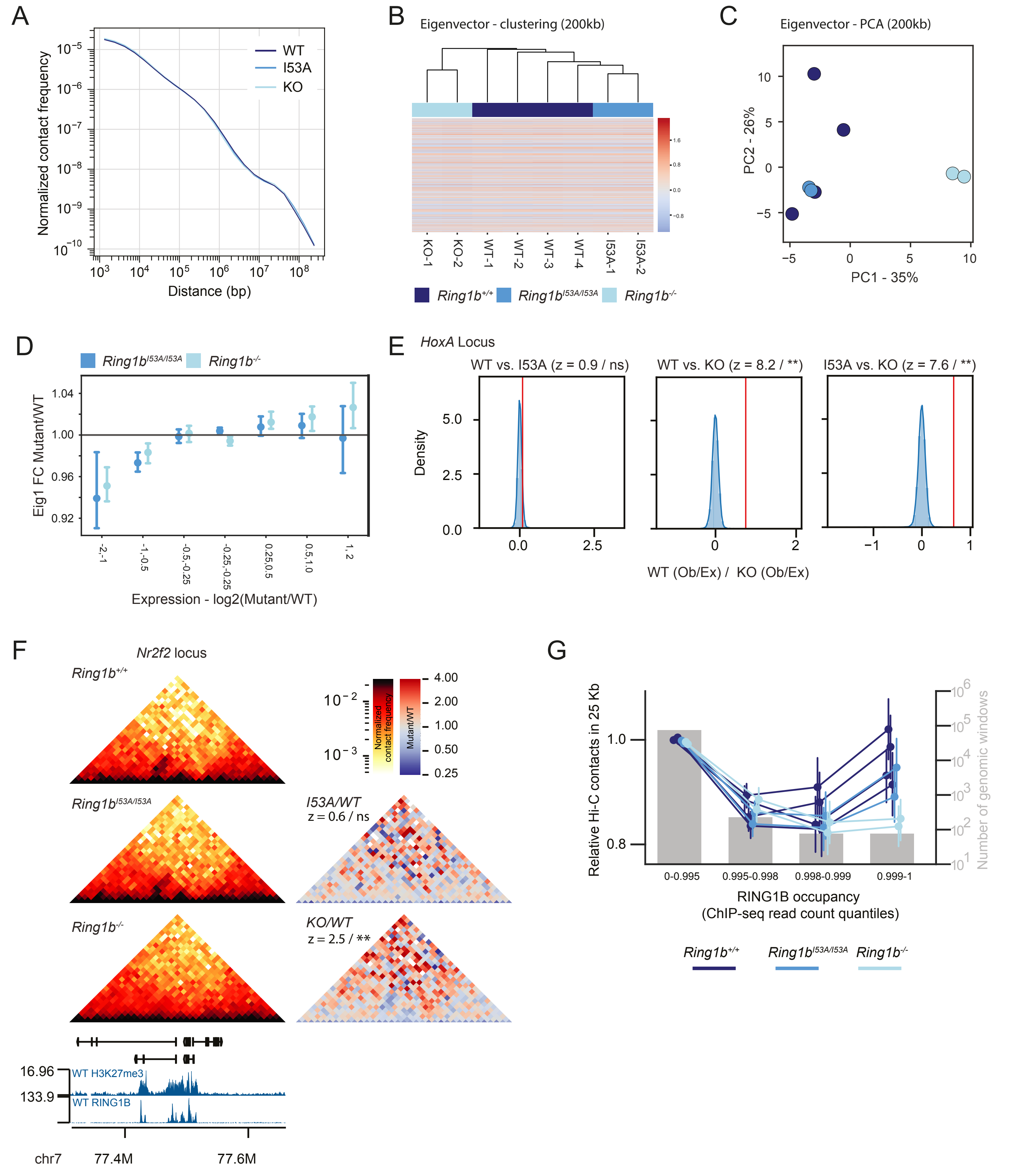

Supplemental Fig. 3. Validation of Hi-C analysis. (A) Curves of probability of contact at different distance separations for $Ring1b^{+/+}$, $Ring1b^{I53A/I53A}$ and $Ring1b^{-/-}$ cells. **(B)** Clustering of first eigenvectors (200 kb resolution) for replicates of Hi-C data for $Ring1b^{+/+}$, $Ring1b^{I53A/I53A}$ and $Ring1b^{-/-}$ mESCs. **(C)** PCA analysis of eigenvectors from **(B)**. **(D)** Median fold-change of first eigenvectors from Hi-C data from $Ring1b^{I53A/I53A}$ and $Ring1b^{-/-}$ mESCs relative to $Ring1b^{+/+}$ mESCs (error bars show 95% confidence interval obtained by bootstrapping), for all 50 kb bins grouped by average log2 fold change in gene expression relative to $Ring1b^{+/+}$ within the bin. **(E)** Statistical estimation of contact frequency changes within the *HoxA* cluster for all pairwise comparisons between $Ring1b^{+/+}$, $Ring1b^{I53A/I53A}$ and $Ring1b^{-/-}$ cells. Shown are histograms of ratios of observed/expected signal for 10,000 random regions on the same chromosome (bars) and its associated kernel-density estimate (curve). The red vertical line represents the value for the *HoxA* region. Z-value and level of significance shown. **(F)** Same as **Fig. 3A**, but for the *Nr2f2* region (statistical estimation performed on chr7 77.43 – 77.52 Mb; mm9 genome build). **(G)** Same as **Fig. 3E**, but for individual replicates.
