# Supplementary material for: A central role for canonical PRC1 in shaping the 3D nuclear landscape
Source: Genes Dev. 2020 Jul 1;34(13-14):931–49. doi: 10.1101/gad.336487.120 (PMC7328521; doi:10.1101/gad.336487.120)
Supplement: Supplemental Material [file supp_gad.336487.120_Supplemental_Fig_4.pdf]

Boyle et al. Supplemental Fig. 4

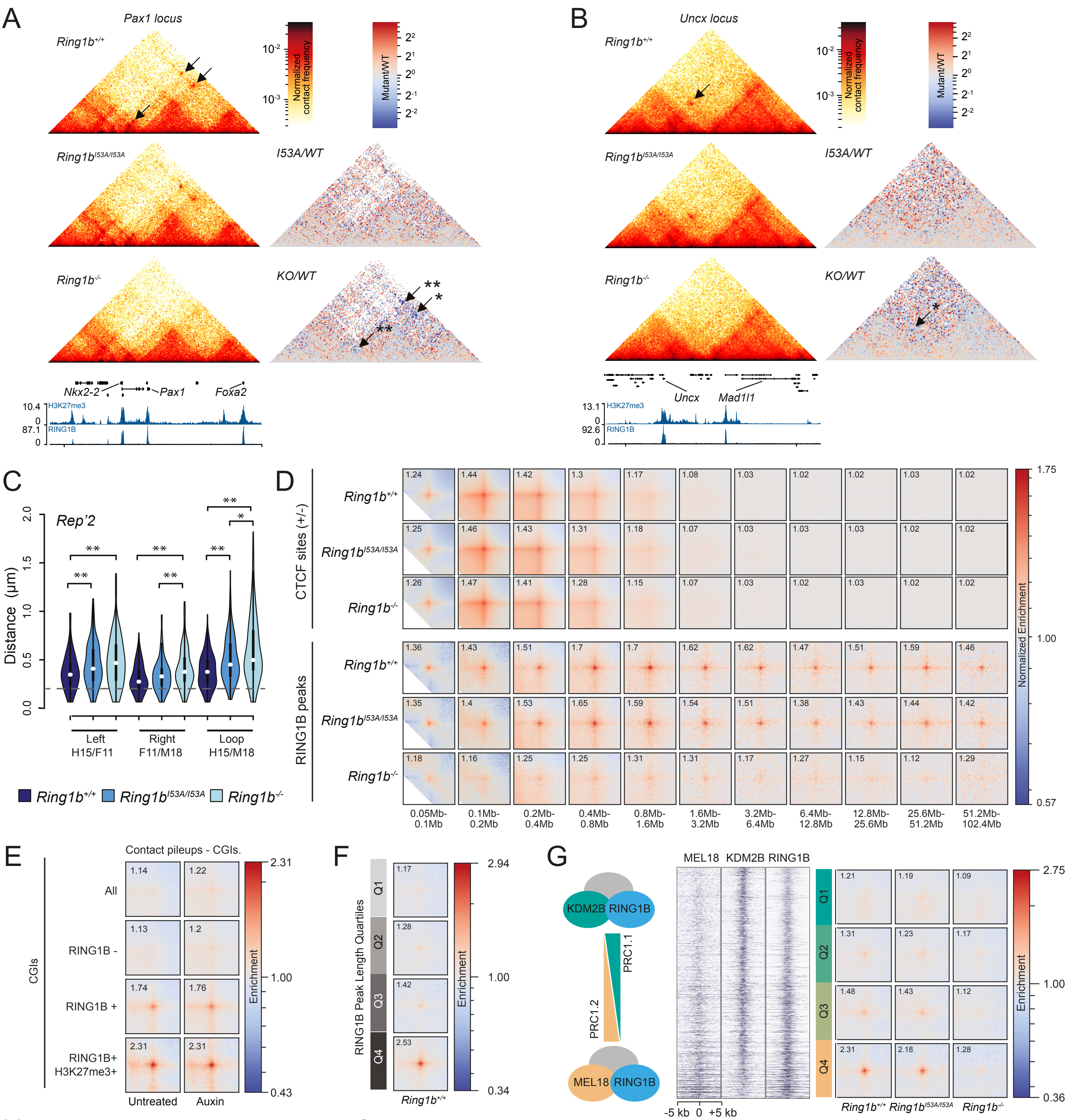

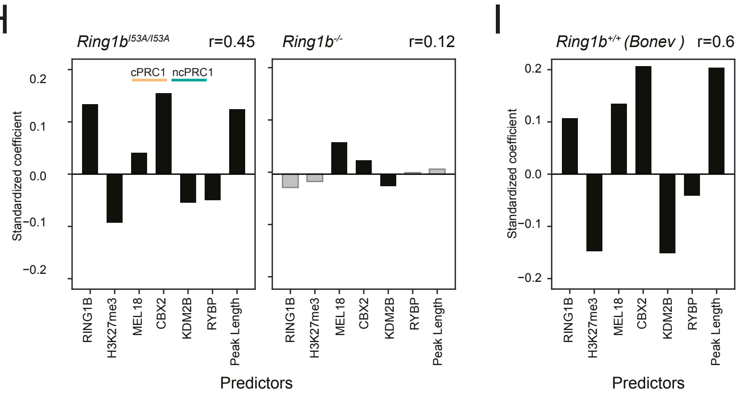

Supplemental Fig. 4. Additional Analysis of Looping Between PRC1 targets. (A) Same as **Fig. 4A**, but for the loops between RING1B peaks proximal to the *Nkx2-2*, *Pax1* and *Foxa2* genes. **(B)** Same as **Fig. 4A**, but for the loops between RING1B peaks proximal to the *Uncx* and *Elfn1* genes. **(C)** Same as **Fig. 4B**, but for two FISH replicates separately. **(D)** Same as for **Fig. 4D**, but for *Ring1b*$^{+/+}$, *Ring1bl*$^{I53A/I53A}$ and *Ring1b*$^{-/-}$ cells and only convergent CTCF sites and RING1B peaks. In rows: convergent CTCF peaks for *Ring1b*$^{+/+}$, *Ring1b*$^{I53A/I53A}$ and *Ring1b*$^{-/-}$ cells, then wildtype RING1B peaks in *Ring1b*$^{+/+}$, *Ring1b*$^{I53A/I53A}$ and *Ring1b*$^{-/-}$ mESCs. **(E)** Same as **Fig. 4C**, but for published Hi-C data from untreated or auxin-treated CTCF-AID cells (Nora et al. 2017). **(F)** Same as **Fig. 4E**, but for peak region length instead of RING1B ChIP-seq signal, and only for *Ring1b*$^{+/+}$ cells. **(G)** Same as **Fig. 4F**, but for MEL18/KDM2B ratio. **(H)** Same as **Fig. 4G**, but for *Ring1b*$^{I53A/I53A}$ and *Ring1b*$^{-/-}$ cells. **(I)** Same as **Fig. 4G**, but for published data from wild-type ES cells (Bonev et al. 2017).
