# Supplementary figures and images for: A central role for canonical PRC1 in shaping the 3D nuclear landscape
Source: Genes Dev. 2020 Jul 1;34(13-14):931–49. doi: 10.1101/gad.336487.120 (PMC7328521; doi:10.1101/gad.336487.120)

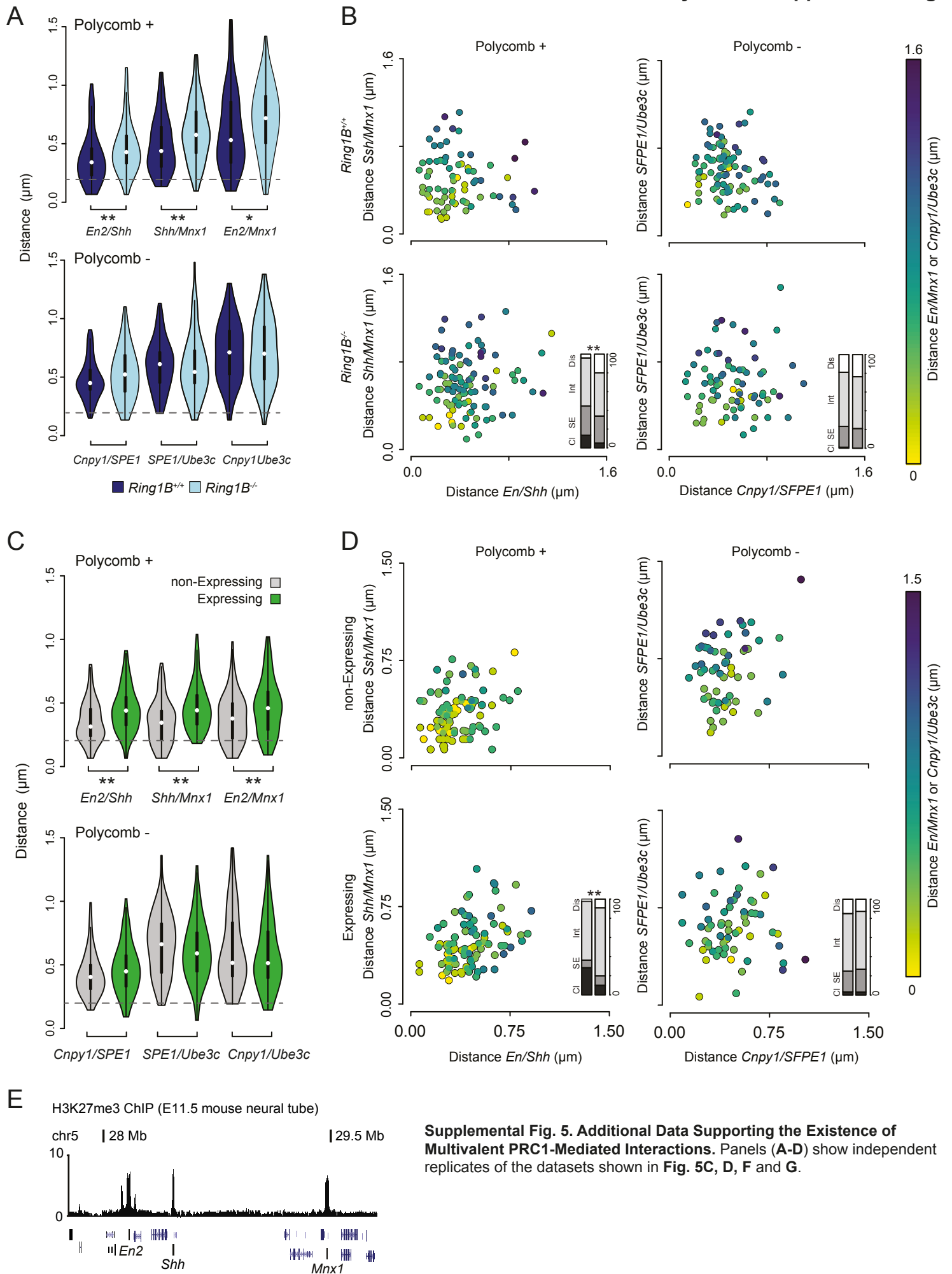

Supplement: Supplemental Material [file supp_gad.336487.120_Supplemental_Fig_5.pdf]
