# Supplementary material for: A central role for canonical PRC1 in shaping the 3D nuclear landscape
Source: Genes Dev. 2020 Jul 1;34(13-14):931–49. doi: 10.1101/gad.336487.120 (PMC7328521; doi:10.1101/gad.336487.120)
Supplement: Supplemental Material [file supp_gad.336487.120_Supplemental_Fig_6.pdf]

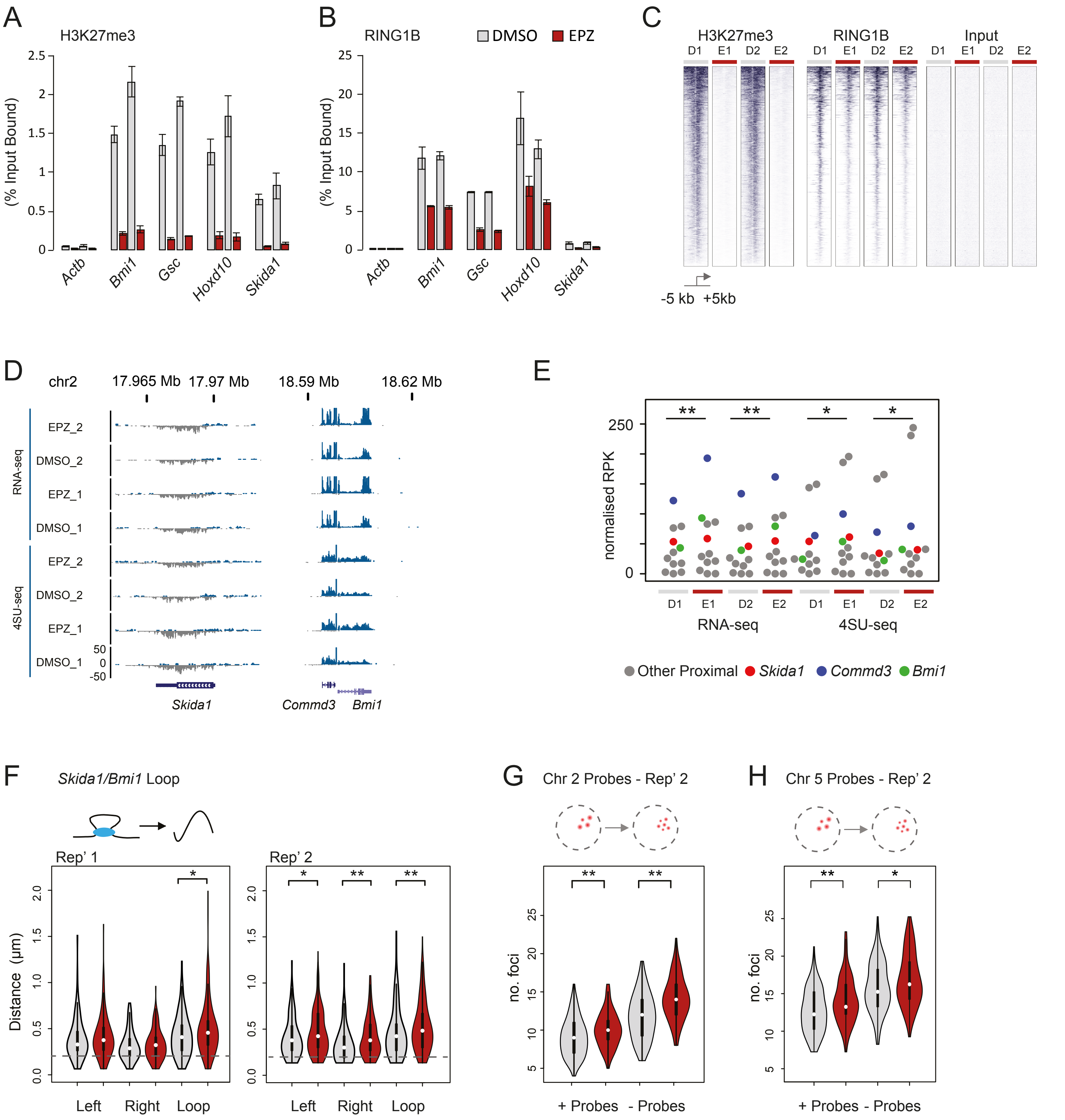

Supplemental Fig. 6. Loss of PRC1 and not Transcriptional Upregulation Results in the Loss of Chromatin Contacts. (**A** & **B**) Barplots representing candidate analysis of ChIP enrichment relative to input measured by quantitative PCR for H3K27me3 (**A**) and RING1B (**B**) in mESCS treated for DMSO or EPZ (two independent replicates are shown). (C) Heatmap representation of H3K27me3 and RING1B ChIP-seq signal distribution at refseq gene TSS (+/- 5 kb; enriched for their respective marks) in two independent sets of EPZ/DMSO treated mESCs. (**D**) Example browser track views of RNA-seq and 4SU-seq data from mESCs following 24 h of EPZ/DMSO treatment (2 independent replicates shown). The signal is coloured according to the transcribed strand (positive - blue and negative -grey). (**E**) Beeswarm plots illustrating the RNA-seq and 4SU-seq signal of genes proximal to *Skida1* / *Bmi1* in mESCs treated with either DMSO or EPZ for 24 h. Significant differential expression is indicated (*p ≤ 0.05 & > 0.01 and **p ≤ 0.01; paired Wilcoxon Rank Sum test). (**F**) 3D FISH measurement for probes shown in (**Fig. 4A**) in mESCs treated with DMSO or EPZ for 24 h (Two independent replicate experiments are shown). The significance of a shift in inter-probe distance between a given pair of samples is indicated (*p ≤ 0.05 & > 0.01 and **p ≤ 0.01; Mann Whitney test). Probes separated by less than 0.2 μm (dashed grey line) are considered to be co-localised. (**G** and **H**) Replicated experiments for those shown in **Fig. 6G** and **H**.
